# Influenza viral matrix 1 protein aggravates viral pathogenicity by inducing TLR4-mediated reactive oxygen species production and apoptotic cell death

**DOI:** 10.1038/s41419-023-05749-5

**Published:** 2023-03-30

**Authors:** Chang-Ung Kim, Dahwan Lim, Young Sang Kim, Bonsu Ku, Doo-Jin Kim

**Affiliations:** 1 Infectious Disease Research Center, Korea Research Institute of Bioscience and Biotechnology (KRIBB), Daejeon, South Korea; 2 Department of Biochemistry, Chungnam National University, Daejeon, South Korea; 3 Disease Target Structure Research Center, Korea Research Institute of Bioscience and Biotechnology (KRIBB), Daejeon, South Korea; 4 University of Science and Technology (UST), Daejeon, South Korea

**Keywords:** Infection, Cell death and immune response, Influenza virus, Acute inflammation

## Abstract

Influenza virus is one of the most challenging viruses threating human health. Since infection with influenza virus triggers inflammatory responses and induces cell death, the molecular and cellular mechanisms by which the virus-infected cells undergo apoptotic and necrotic cell death have been widely studied. However, most of the studies have focused on the molecular events occurring in the cytosol and there is limited information on the physiological correlation between virus-induced cell death and the viral pathogenesis in vivo. In this study, we demonstrate that the influenza virus matrix 1 (M1) protein is released from virus-infected cells and triggers apoptotic cell death of lung epithelial and pulmonary immune cells, through the activation of Toll-like receptor 4 (TLR4) signaling. Treatment with M1 protein led to robust cellular inflammatory responses, such as the production of proinflammatory cytokines and cellular reactive oxygen species (ROS), and induction of cell death. When M1 protein was administered in vivo, it induced the activation of inflammatory responses and cell death in the lungs. Furthermore, the administration of M1 aggravated lung pathology and mortality of the virus-infected mice in a TLR4-dependent manner. These results demonstrate that M1 is an important pathogenic factor contributing to influenza virus pathogenicity by enhancing cell death in the lungs, thereby expanding our understanding of the molecular mechanism of influenza virus-induced cell death through the interaction with an innate immune receptor.

## Introduction

Influenza is an acute respiratory viral disease with approximately 3 to 5 million cases of severe illness and up to 650000 deaths annually, worldwide. Therefore, the underlying mechanisms of the pathogenesis of the influenza virus have attracted extensive attention. Previous reports have demonstrated that influenza virus triggers various inflammatory responses, such as the upregulation of proinflammatory cytokines and chemokines, overproduction of intracellular reactive oxygen species (ROS), and enhanced programmed cell death [1–3]. Since these cellular responses can lead to severe lung injury, identification of precise inflammation-associated factors and pathways is important for understanding the pathogenesis of influenza and developing novel therapeutic strategies.

Acute viral infection is commonly associated with cell death. Since lung epithelial and pulmonary immune cells directly infected with influenza virus undergo apoptotic, necrotic and necroptotic cell death, the underlying molecular mechanisms have been widely studied [4–7]. Particularly, intracellular interactions between viral and host proteins have attracted the most attention because it has been regarded that viral proteins, especially internal proteins, are expressed and reside in the cytosol. However, it was recently reported that nucleoprotein (NP) can be released from infected cells and increase viral pathogenicity through the activation of Toll-like receptor 4 (TLR4), a membrane receptor, on innate immune cells in the lungs [8]. This suggests a potential role for viral internal proteins in the disease pathogenesis through the interaction with host cells in the extracellular compartment.

Influenza virus matrix 1 (M1) protein is important for viral replication and shaping of virus particles [9]. M1 also interacts with cytosolic host proteins such as caspase-8 and heat shock protein 70 to accelerate apoptosis of the infected cells [10, 11]. However, despite extensive studies at the molecular and cellular levels, the physiological role of M1 in viral pathogenicity in vivo has not yet been comprehensively addressed. In this study, we investigated the role of influenza virus M1 protein in cell death and viral pathogenicity in vivo. Our results showed that extracellular M1 triggered apoptotic cell death of the lung epithelial cells and pulmonary immune cells through the activation of TLR4 signaling pathway and the production of ROS in vitro and in vivo. Our findings indicate that extracellular M1 is an important pathogenic factor contributing to influenza virus pathogenicity by enhancing cell death in the lungs, thereby expanding our understanding of the molecular mechanism of influenza virus-induced cell death.

## Results

### Extracellular M1 activates intracellular inflammatory pathways

**Fig. 1:**
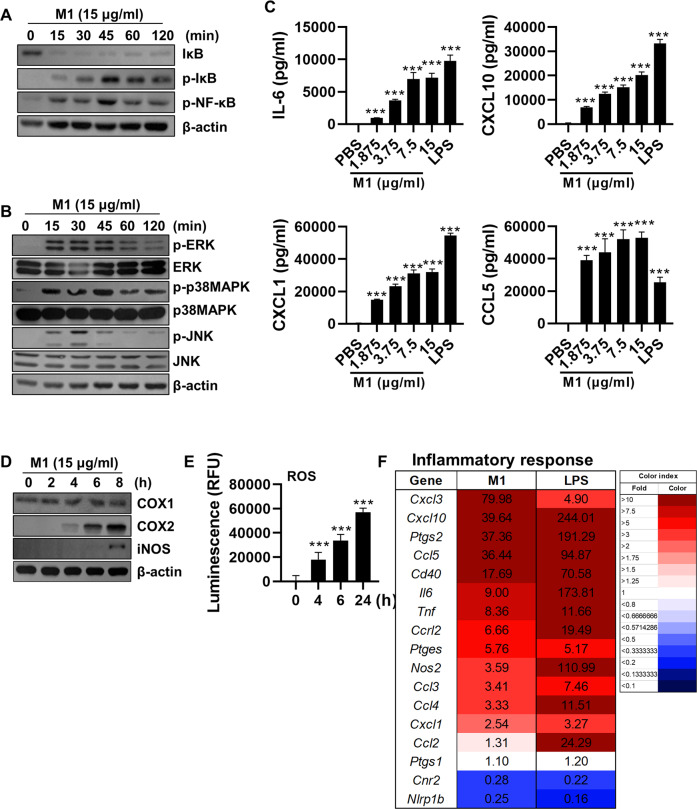
Extracellular treatment with M1 provokes inflammatory responses. BMDMs were treated with rM1 and analyzed as described in the text. Activation of the (**A**) NF-κB and (**B**) MAPK pathways were detected at the indicated points in time by immunoblotting. **C** After 24 h of treatment with various concentrations of rM1, cell culture supernatants were used for ELISA to detect the levels of the indicated cytokines and chemokines (*n* = *5*). **D** Levels of proteins involved in the ROS production were traced at each time point by immunoblotting. **E** ROS levels were detected in cell culture supernatants of cells treated with rM1 (15 μg/ml) (*n* = *5*). **F** The inflammatory response genes regulated by M1 and LPS treatment was analyzed by RNA-sequencing is presented as a heatmap (*n* = *2*). Data are presented as the mean ± standard deviation (SD) from triplicate culture wells. ***p < 0.001.

rM1 was produced using the CHO cell expression system (Fig. S1A, B) and was confirmed to be endotoxin-free (Fig. S1C). When bone marrow-derived macrophages (BMDMs) were treated with rM1, nuclear factor-kappa B (NF-κB) pathway and mitogen-activated protein (MAP) kinase family members were activated within 15 min (Fig. 1A, B). Consequently, the production of proinflammatory cytokines and chemokines, such as IL-6 and C-X-C motif ligand (CXCL10), was remarkably upregulated (Fig. 1C). Furthermore, time-dependent enhanced expressions of peroxide synthases like COX2 and iNOS were also observed (Fig. 1D), which accounts for the increased production of ROS (Fig. 1E). In addition, the increase in the inflammatory response including proinflammatory cytokines, chemokines, and ROS-regulated genes owing to M1 treatment was confirmed via RNA sequencing analysis (Fig. 1F). Similarly, rM1 treatment led to enhanced expression of proinflammatory cytokines and chemokines and upregulation of ROS in murine lung epithelial (MLE)-12 (Fig. S2A, B) and bronchoalveolar lavage (BAL) cells (Fig. S2C, D). Collectively, these data demonstrated that extracellular M1 treatment can induce intracellular inflammatory pathways in host cells.

### TLR4 plays a key role in M1-induced inflammatory responses

**Fig. 2:**
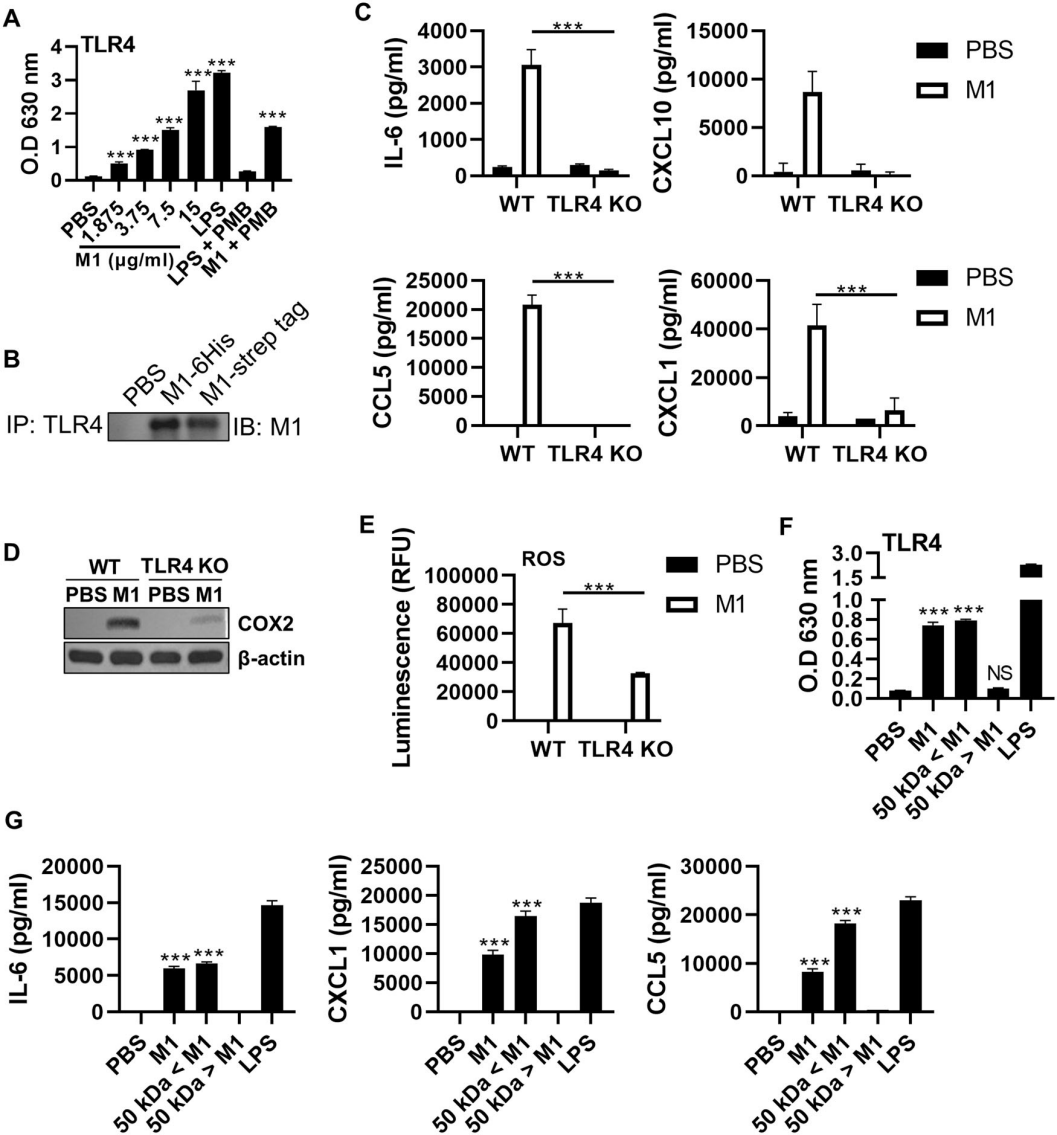
Inflammatory functionality of M1 depends on TLR4. **A**, **F** TLR4 reporter and (**B**–**E** and **G**) BMDM cells were treated with rM1 (**A** and **F**, as indicated; **B**–**G**, 15 μg/ml) and analyzed as described in the text (**A**, **C**, and **E**–**G**; *n* = *5*). (A) TLR4 reporter cells were incubated with the indicated materials for 20 h, and the TLR4 expression level was analyzed by measuring OD_630_. **B** After 30 min of treatment with the two types of rM1, IP was performed to detect interactions between TLR4 and M1. **C** After 24 h of rM1 treatment, wild-type (WT) and TLR4^−/−^ cell culture supernatants were subjected to ELISA to determine the levels of indicated cytokines and chemokines. **D** The effects of rM1 treatment (8 h) on COX2 levels in WT and TLR4^−/−^ cells were measured by immunoblotting. **E** ROS levels detected in WT and TLR4^−/−^ cell culture supernatants. **F** TLR4 reporter cells were incubated with the indicated materials for 20 h; thereafter, OD_630_ was measured. **G** After 24 h of incubation with the indicated materials, cytokines and chemokines were detected in cell culture supernatants. Data are presented as the mean ± SD from triplicate culture wells. ***p < 0.001.

Next, we sought to identify the host receptor responsible for recognizing M1 and initializing inflammatory responses. We found that the upregulation of IL-6 expression and ROS production, usually observed in TLR4-mediated signaling processes, was observed in rM1-treated BMDMs (Fig. 1C, E), MLE-12, and BAL-derived cells (Fig. S2). Moreover, inactivated influenza virus triggers the production of oxidized phospholipid via the TLR4 − IL-6−ROS signaling cascade, which causes acute lung injury [12]. Therefore, we hypothesized that TLR4 plays a key role in the rM1-induced inflammatory responses. Dose-dependent TLR4-stimulating activity of rM1 was detected when TLR4-expressing reporter cells were treated with rM1 (Fig. 2A). A subsequent immunoprecipitation (IP) assay revealed a physical interaction between rM1 and TLR4 in rM1-treated BMDM cell lysates (Fig. 2B). Specifically, genetic ablation of TLR4 in BMDM cells led to a significant impairment of rM1-induced production of proinflammatory cytokines, including IL-6, CXCL1, CXCL10, and C-C motif ligand (CCL)5 (Fig. 2C). Furthermore, COX2 peroxide synthase expression (Fig. 2D) and ROS (Fig. 2E) levels were remarkably reduced in TLR4-deficient cells.

It has been reported that several viral proteins can activate TLR4 [13]. Recently, it was proposed that the multimeric or oligomeric structure of viral proteins is important for TLR4 activation [8, 13]. Although oligomeric M1 is abundant, monomeric M1 also exists at equilibrium in vitro and in vivo (Fig. S3A, B) [9, 14, 15]. To determine whether the stoichiometry of M1 affects its functionality toward TLR4, we separated M1 into two fractions of less than and greater than 50 kDa (Fig. S4A, B). Only the M1 fraction of >50 kDa induced TLR4 activation, whereas monomeric M1 did not (Fig. 2F). Proinflammatory cytokines and chemokines were also induced only by oligomeric M1 (Fig. 2G). These results indicate that M1, as an oligomeric form, induces the inflammatory response through the activation of TLR4.

### M1 induces cell death via ROS production and caspase cascades

**Fig. 3:**
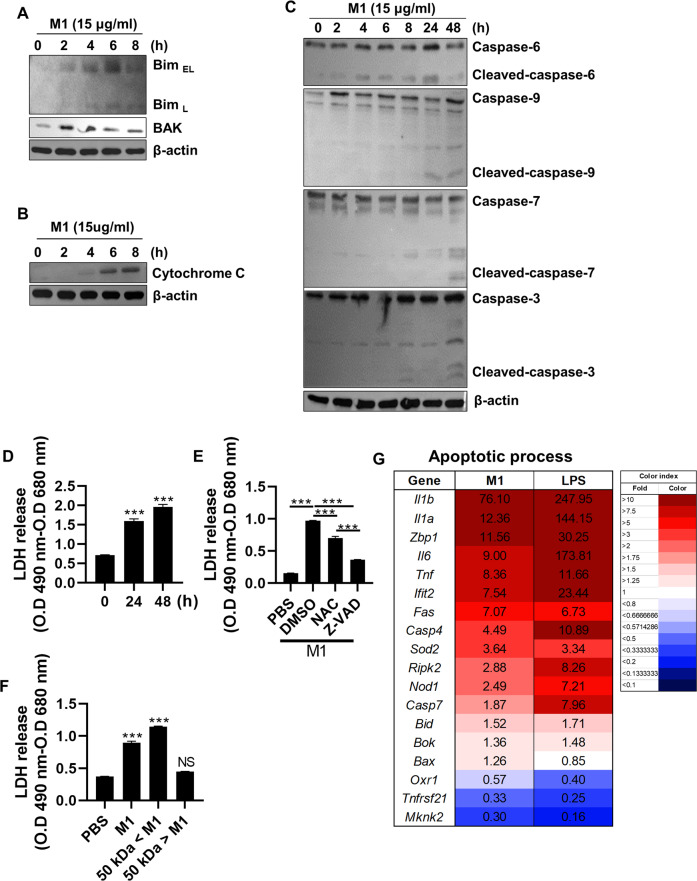
M1 activates apoptotic pathways and induces cell death. BMDMs were treated with rM1 (15 μg/ml) and analyzed as described. **A** Levels of pro-apoptotic factors, (**B**) cytochrome *c*, and (**C**) cleaved caspases were determined at the indicated time points by immunoblotting. **D** LDH levels detected in the cell culture supernatants at indicated time point (*n* = *5*). **E**–**F** LDH levels detected in the cell culture supernatants after 24 h of incubation with the indicated materials (*n* = *5*). **G** The apoptotic process regulated at gene level by M1 and LPS treatment was analyzed by RNA-sequencing and is presented as a heatmap (*n* = *2*). Data are presented as the mean ± SD from triplicate culture wells. ***p < 0.001.

Given that excessive ROS increase mitochondria damage, which induces apoptotic cell death [16, 17], we next examined whether rM1 treatment also affects cell fate via upregulation of ROS production (Figs. 1E and 2E). We found a prominent increase in the protein levels of pro-apoptotic factors such as Bim_EL_, Bim_L_, and Bak in BMDM cells upon rM1 treatment (Fig. 3A). Increased cytochrome C levels were detected in the cytosol upon M1 administration, indicating mitochondrial damage (Fig. 3B). Consistently, rM1 treatment induced cleavage of caspase proteins, including caspase-3, −6, and −7, which are characteristic markers of activation of the caspase cascades that mediate apoptosis (Fig. 3C). Consequently, rM1 treatment caused the death of BMDM cells in a time-dependent manner, which was verified by the lactate dehydrogenase (LDH) release assay (Fig. 3D). Furthermore, cell death was attenuated by treatment with the ROS inhibitor N-acetyl-l-cysteine (NAC) or pan-caspase inhibitor Z-VAD-FMK (Fig. 3E). Consistent with the result of cytokine production, oligomeric M1, not monomeric M1, induced apoptotic cell death (Fig. 3F). In addition, M1-treated BMDMs had increased mRNA levels of factors involved in apoptotic processes such as *Tnf, Fas, Bid, and Bax* (Fig. 3G). Similarly, rM1 treatment induced the death of MLE-12–and BAL-derived cells (Fig. S5A, B). These results indicate that oligomeric M1 triggers apoptotic cell death via ROS production and a caspase cascade activation-dependent pathway.

### M1 activates pulmonary immune cells and induces inflammatory responses in the lungs

**Fig. 4:**
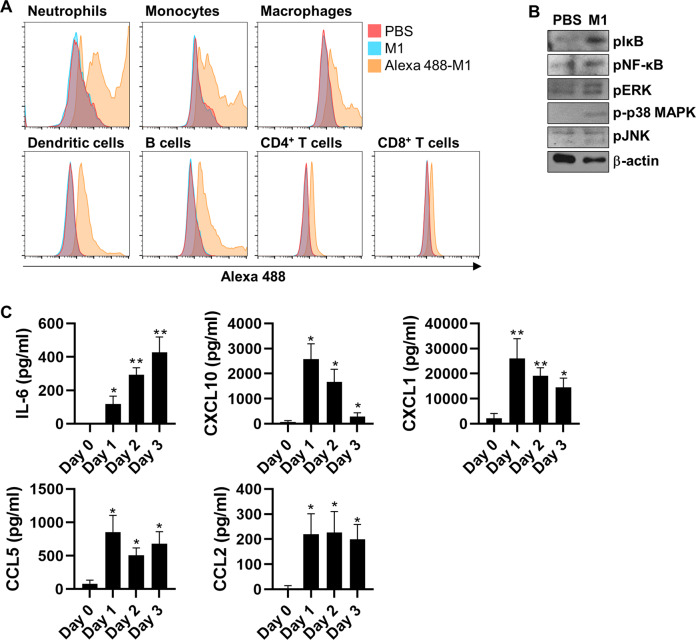
M1 recognizes pulmonary immune cells and activates inflammatory responses in mouse lungs. **A** Cells isolated from mouse lungs were treated with rM1 or Alexa 488-labeled rM1 (15 μg/ml) for 15 min and then analyzed by flow cytometry to determine the M1–cell association (*n* = *5*). **B** Mice were intranasally treated with PBS or rM1 (15 μg/ml) for 6 h (*n* = *5*). BAL fluid cells were isolated from these mice and subjected to immunoblotting to detect the activation of NF-κB and MAPK signaling. **C** Mice were intranasally treated with PBS or rM1 (30 μg/ml) for 0–3 days. BAL fluid was extracted from these mice and subjected to ELISA every day to measure the levels of the indicated cytokines and chemokines (*n* = *5*). Data are presented as mean ± standard error of the mean (SEM). **p < 0.01, *p < 0.05.

After demonstrating that cellular inflammatory responses and ROS-associated apoptosis can be induced by extracellular treatment with a recombinant form of influenza virus M1 protein (Figs. 1–3), we attempted to verify the functionality of M1 in vivo using a mouse model. First, to identify which cell types are mainly activated by M1, we isolated various immune-associated cells from mouse lung tissues and incubated them with Alexa Fluor 488–labeled rM1 for 15 min. A flow cytometry analysis showed that Alexa Fluor 488-conjugated rM1 was vividly detected in neutrophils, monocytes, and macrophages, which are key players in innate immune responses (Fig. 4A). By contrast, it was weakly detected in cells controlling adaptive immune responses, such as B and T cells (Fig. 4A). Next, we intranasally treated the mice with rM1 and analyzed their response. Activation of NF-κB and MAPKs were identified in BAL-isolated cells 6 h after rM1 treatment (Fig. 4B). The protein levels of proinflammatory cytokines and chemokines in BAL fluid also increased noticeably 1–3 days after intranasal treatment with rM1 (Fig. 4C).

**Fig. 5:**
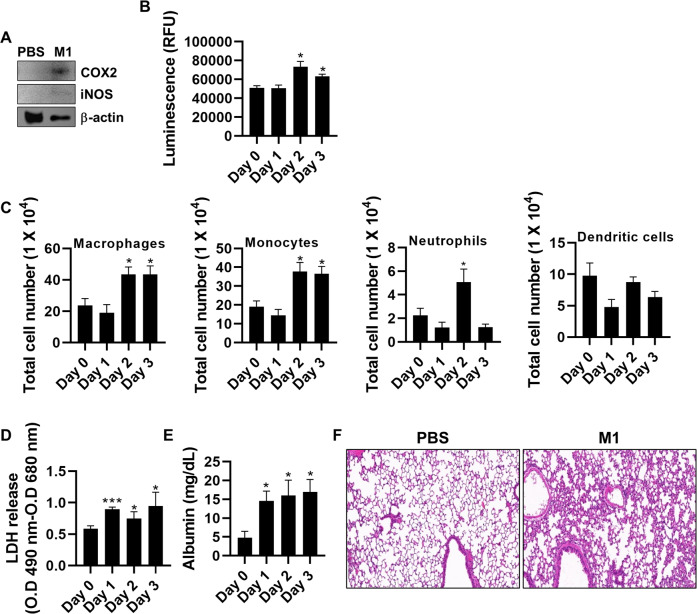
M1 increases lung damage through ROS production and apoptosis. **A** Mice (*n* = *5*) were intranasally treated with PBS or rM1 (30 μg) for 6 h. BAL cells were isolated and subjected to immunoblotting for detecting COX2 and iNOS. **B**–**F** Mice were intranasally treated with PBS or rM1 (30 μg) for 0–3 days (**B**) BAL fluid was extracted, followed by the in vitro ROS/RNS assay for ROS measurement. **C** Total lung tissue was isolated and subjected to surface staining and intracellular iNOS staining for flow cytometry analysis. **D** LDH levels detected in BAL fluid using the LDH cytotoxicity assay (**B**–**D**, *n* = *5*). **E** Albumin levels in BAL fluid detected using the BCG albumin assay kit. **F** Hematoxylin and eosin (H&E) staining was used to visualize lung tissues obtained on day 3 (*n* = *2*). Data are presented as the mean ± SEM. ***p < 0.001, **p < 0.01, *p < 0.05.

Next, we analyzed whether ROS production and the consequent cell death were upregulated by intranasal treatment with rM1. Activation of COX2 and iNOS was observed in BAL-derived cells (Fig. 5A), which led to the upregulation of ROS levels in the BAL fluid (Fig. 5B), in rM1-treated mice after 6 h. We also noticed that iNOS levels were remarkably enhanced in macrophages, monocytes, and neutrophils, cell types that were majorly targeted by rM1 (Fig. 4A). However, iNOS levels were not enhanced in dendritic cells even 2 days after rM1 treatment (Fig. 5C). The LDH level in BAL fluid was elevated upon rM1 treatment, implying increased cell death in the lung (Fig. 5D). Subsequently, we hypothesized that the pathology occurs in the rM1-treated mouse lung tissues. In accordance with this hypothesis, the albumin level in BAL fluid was considerably higher after intranasal treatment with rM1 (Fig. 5E), which was also supported by the histological analysis of rM1-treated mouse lung tissues (Fig. 5F). These results suggest that influenza virus M1 protein targets pulmonary immune cells in the lungs and induces inflammatory responses, such as cytokine production and ROS generation, in the lungs.

### M1 aggravates influenza pathogenicity in a TLR4-dependent manner

**Fig. 6:**
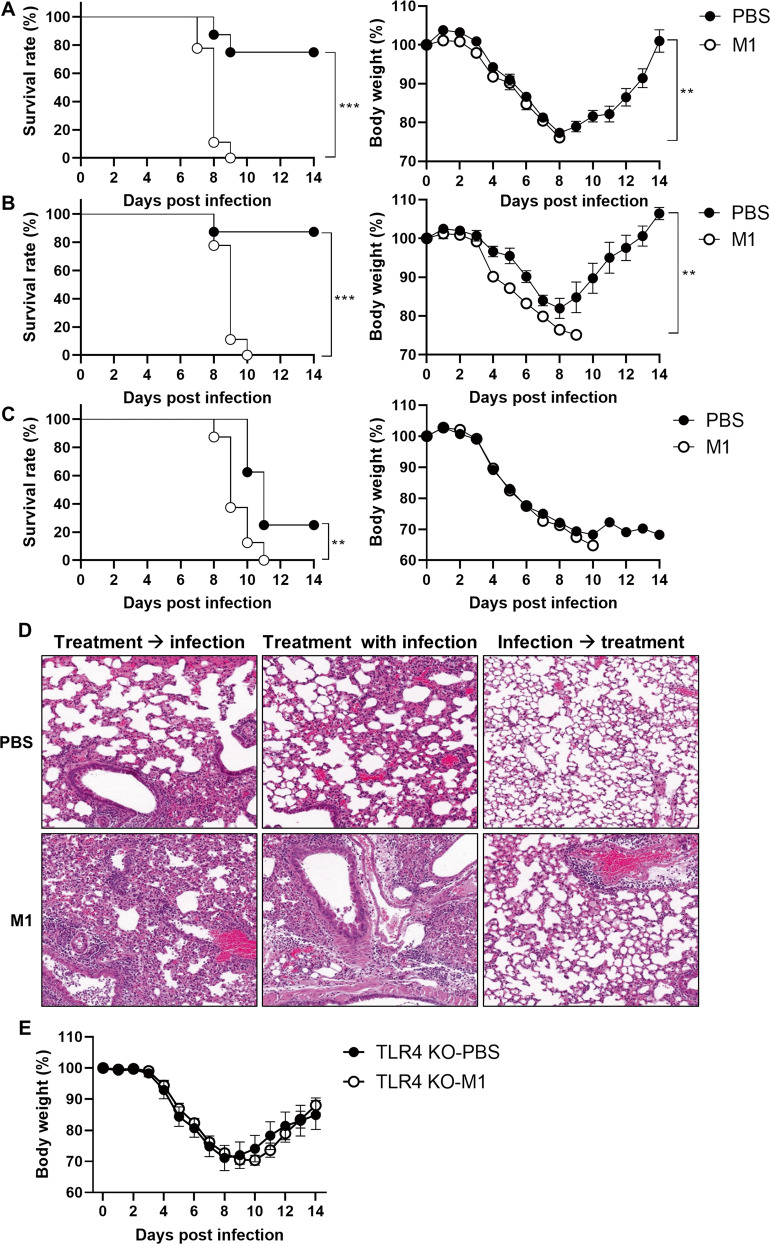
M1 aggravates influenza pathogenesis in a TLR4-dependent manner. Mice were intranasally treated with rM1 (30 μg) 3 days prior to (**A** and left panel of **D**) or simultaneously with (**B** and middle panel of **D**) or 3 days after (**C** and right panel of **D**) PR8 influenza infection. **A**–**C** Survival rates and body weight change were monitored for 2 weeks (*n* = *8*). **D** Lung tissues obtained on day 5 post infection were visualized using H&E staining (*n* = *2*). **E** TLR4^−/−^ mice were treated intranasally with rM1 (30 μg) and co-infected with PR8 influenza virus (32 PFU), and the body weight change was monitored for 2 weeks (*n* = *8*). ***p < 0.001, **p < 0.01, *p < 0.05 in the log-rank test.

Next, we investigated whether M1 protein affects influenza pathogenicity. Mice were intranasally treated with rM1 (30 μg/mouse) 3 days before (Fig. 6A) or during (Fig. 6B) infection with a sublethal dose (32 PFU) of influenza virus. Remarkably, all rM1-injected mice died within 10 days of infection (Fig. 6A, B, left panels). In contrast, more than 80% of the control group members survived (Fig. 6A, B, left panels) and recovered 8 days post infection (Fig. 6A, B, right panels). In addition, when influenza virus-infected mice were treated with rM1 3 days post infection, the fatality rate of the rM1-treated group was considerably higher than that of the PBS-treated group (Fig. 6C). Further, rM1 treatment severely worsened lung damage in influenza virus-infected mice. These results indicated that rM1 substantially aggravates the pathogenicity of influenza infection. (Fig. 6D). We propose that TLR4 plays a pivotal role in rM1-mediated influenza pathogenicity because no significant difference was observed between TLR4-deficient mice and the control group upon rM1 administration (Fig. 6E). Collectively, these results show that M1 contributes to viral pathogenicity and aggravates lung damage caused by influenza infection, in which TLR4 serves as a critical component.

### Neutralization of M1 effectively diminishes influenza severity

**Fig. 7:**
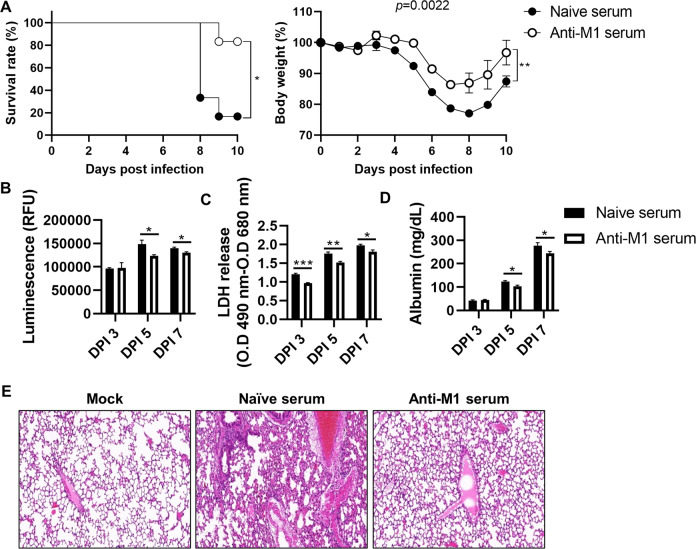
Anti-M1 serum alleviates influenza viral pathogenicity. Mice infected with PR8 influenza virus (32 PFU) were treated with naïve or anti-M1 serum (200 μl) once daily for 3 days. **A** Survival rates and bodyweights of the mice were monitored for 10 days (*n* = *8*). **B**–**D** BAL fluid was extracted on days 3, 5, and 7 post infection and used to analyze levels of (**B**) ROS, (**C**) LDH, (**D**) and albumin. **E** Lung tissues obtained on day 5 were visualized using H&E staining (*n* = *2*). Data in (**A**) are presented as log-rank tests. Data in (**B**–**D**) are presented as mean ± SEM. ***p < 0.001, **p < 0.01, *p < 0.05.

Having shown that M1 plays an important role in influenza virus pathogenesis, we next investigated whether anti-M1 antibodies can counteract the effects of this viral protein. Even though the anti-M1 serum was unable to neutralize the influenza virus in vitro (Fig. S6A), it positively affected the survival rate and body weight of influenza-infected mice that were intraperitoneally treated with anti-M1 serum daily for 3 days (Fig. 7A). Moreover, the fatality of virus-infected mice decreased significantly by injecting purified anti-M1 IgG (Fig. S6B). We also found that anti-M1 serum administration led to a reduction in ROS and LDH levels in the BAL fluid derived from infected mice (Fig. 7B, C), indicating that lung inflammation was suppressed by anti-M1 serum injection. Particularly, lung injury was noticeably alleviated upon anti-M1 serum administration, as demonstrated by the downregulated albumin level in BAL fluid (Fig. 7D) and histological analysis of lung tissues (Fig. 7E). Overall, these results suggest that M1 could be a target for mitigating the pathogenesis of influenza virus infection.

## Discussion

Influenza virus is one of the major pathogens threatening human health worldwide. Since acute viral infection is associated with cell death and tissue damage, the molecular and cellular mechanisms by which the virus-infected cells undergo cell death have been intensively studied. However, most of the studies to date have focused on the interactions between viral components and host proteins in the cytosol of the infected cells [9], where most influenza viral proteins have been regarded to linger. It is also still unclear whether the interactions studied at molecular level affect the disease pathology in vivo. This study aimed to investigate whether and how the influenza viral extracellular M1 protein induces apoptotic cell death in the lungs and affects the viral pathogenesis using a murine influenza model.

Infection with influenza virus primarily induces apoptotic, necrotic, and necroptotic death of lung epithelial and pulmonary immune cells [4–7]. In addition to direct infection, viral infection can also mediate “extrinsic” apoptotic cell death of uninfected cells through the induction of TNF-related apoptosis-inducing ligand (TRAIL) and Fas ligand (FasL) in monocyte-derived macrophages and lymphocytes [18–20]. Here, we showed that M1 is released from the infected cells and induces inflammatory response and apoptotic cell death (Figs. 3 and 5), suggesting that it can also paracrinally affect uninfected cells similar to the death receptor ligands. Consequently, infiltrating immune cells are exposed to proapoptotic environment, implying that the release of M1 could be one of the immune-evading strategies of the virus. Our findings further expand molecular and physiological aspects of the extrinsic cell death and immune evasion mechanism of influenza virus, utilizing a viral protein.

Influenza viral proteins have been generally regarded to be localized inside the infected cells or on the plasma membrane. M1 is localized just beneath the plasma membrane where it forms a mature virion. However, we recently reported that various influenza viral proteins can be detected in a cell-free or virion-free form by viral infection, suggesting the potential of interaction between viral proteins and neighboring cells [8]. In this study, we showed that M1 has a novel biological function in the extracellular compartment through the activation of TLR4 signaling. Although we did not investigate the molecular mechanism by which M1 is released from the infected cells in this study, the possible mechanisms include the following. (i) Infection with influenza virus directly triggers necrotic and necroptotic cell death [7, 21–23], resulting in the leakage of cell contents. M1 can also be exposed to the extracellular compartment during apoptosis. (ii) When the apoptized cells are not completely cleared, the debris could undergo secondary necrosis [24], resulting in the release of internal materials. (iii) In addition, M1 physically interacts with phosphatidylserine of the plasma membrane [25]. The flipping of phosphatidylserine from the inner side to outer side during apoptosis is a possible mechanism of M1 release. Considering that lytic cell death is a common outcome of acute viral infection, further study is required to ascertain the biological function of viral internal proteins in viral pathogenesis through interaction with neighboring cells.

Thus far, activation of pattern recognition receptors (PRRs) by influenza virus has been widely studied at molecular and cellular levels. In many cases, however, it remains unclear whether the PRR activation affects host defense in vivo. Activation of TLR7 and RIG-I by influenza virus infection triggers innate immune responses such as the production of type I IFNs and proinflammatory cytokines result in overall protection in vivo [26–29]. It is also controversial whether the activation of the NLRP3 inflammasome and production of IL-1β and IL-18 increases or decreases lung pathology [30–32]. In this work, we demonstrated that TLR4 recognizes extracellular M1 and aggravates viral pathogenicity in vivo. Interestingly, inactivated H5N1 virus induced acute lung injury in a TLR4-dependent manner, partially supporting our findings [12]. Furthermore, we recently reported the pathogenic role of TLR4 through the interaction with influenza virus NP [8]. Collectively, TLR4 plays a pathogenic role in the pathogenesis of influenza by recognizing a wide range of ligands from oxidized phospholipid to viral proteins.

Excess levels of ROS can lead to mitochondrial damage and consequent apoptotic cell death. In the case of influenza viral infection, oxidative stress by ROS provokes inflammation and acute lung injury [12, 33, 34]. It is well known that oxidized phospholipid can trigger ROS production through the activation of TLR4 [12]. In this study, we additionally showed that M1, the most abundant influenza viral protein, can be another trigger for ROS production in vitro and in vivo. However, it is intriguing that cellular ROS is also required for the optimal induction of the antigen-specific CD8^+^ T cell response by increasing antigen cross-presentation by dendritic cells [35–37]. In our study, we did not investigate the effect of M1 on the fate or behavior of dendritic cells. It is worthwhile to study whether and how M1 differentially modulates the host response to the viral infection depending on physiological conditions such as its micro-concentration and the cell types it encounters.

The activation of TLR4 has different sub-signaling mechanisms depending on the type of ligand [38, 39]. Bacteria-derived LPS activates both MyD88-dependent and TRIF-dependent pathways [38], but monophosphoryl lipid A (MPLA), a low-toxic derivative of lipid A moiety of LPS, predominantly activates the TRIF-dependent pathway [40, 41]. Consequently, TLR4 activation by LPS and MPLA differs in the induction of transcriptome change, chemokine production, inflammation, and memory CD8^+^ T cell differentiation [42, 43]. Thus, identifying novel ligands of TLR4 and their biological consequence is of continuing interest. Our gene expression profile results showed that M1 and LPS differentially activated downstream target genes, and, interestingly, it did not match those prompted by NP (Figs. 1F and 3F, and Fig. S7A–C). Therefore, further study is required on ligand dependent TLR4 signaling mechanisms in terms of molecular and structural biology.

Previous studies have demonstrated that anti-M1 antibody cannot directly neutralize influenza virus [44]. However, our data showed that anti-M1 antibody alleviates disease severity and improves survival rate against the viral challenge. Anti-M1 plasma or anti-M1 antibody reduced the level of inflammatory cytokines and cell death in infected mice. Antibodies to NP have shown an antiviral effect against influenza virus infection in murine models [45–47]. Although it has been previously suggested that NK cells contribute to the antiviral effect via antibody-dependent cellular cytotoxicity, the exact mechanism has not been experimentally proved. In this study, we did not investigate the possibility of involvement of NK cells in the anti-M1 antibody-mediated therapeutic effect. Nonetheless, antibodies targeting M1 could be an auxiliary therapeutic approach for influenza.

In conclusion, we showed that M1 is an important pathogenic factor contributing to influenza viral pathogenicity by increasing ROS-mediated cell death in the lungs. Intriguingly, M1 is released from infected cells to the extracellular compartment and interacts with TLR4, a membrane receptor protein, on the lung epithelial and pulmonary immune cells. This finding not only expands our understanding of the molecular mechanism of influenza virus-induced cell death and viral pathogenesis but could also be useful information for the development of a novel antiviral drug.

## Materials and methods

### Mice

Seven- to eight-week-old C57BL/6 and BALB/c female mice were purchased from KOATECH (Pyeongtaek, Korea) and maintained in a specific pathogen-free biosafety level-2 facility at the Korea Research Institute of Bioscience and Biotechnology (KRIBB). TLR4 knockout mice were purchased from The Jackson Laboratory (Bar Harbor, Maine, USA). Only 8- to 12-week-old female mice were used in this study. All animal experiments were approved by the Institutional Animal Use and Care Committee of KRIBB (KRIBB-AEC-19173) and performed in accordance with the Guide for the Care and Use of Laboratory Animals published by the US National Institutes of Health.

### Cell culture

HEK-Blue™ TLR4 (InvivoGen, San Diego, CA, USA) and L929 (ATCC, Manassas, Virginia, USA) cells were maintained in Dulbecco’s Modified Eagle Medium (DMEM, Corning, NY, USA) supplemented with 10% fetal bovine serum (FBS, HyClone, Logan, Utah, USA) and 1× antibiotics (Gibco™, Massachusetts, USA). MLE-12 cells (ATCC) were maintained in DMEM supplemented with 2% FBS and 1× antibiotics. No mycoplasma contamination was detected. To prepare BMDMs, bone marrow cells were harvested from the femur and tibia of C57BL/6 N and TLR4 knockout mice and differentiated in DMEM containing 25% L929-conditioned medium, 10% FBS, and 1× antibiotics. L929-conditioned medium was obtained by culturing L929 cells in DMEM for 7 days until a confluence of >90% was obtained. Afterwards, the medium underwent centrifugation at 1500 rpm for 10 min and the supernatants were collected and filtered through a 0.45 μm filter system.

### Recombinant M1 protein

The DNA fragment encoding M1 protein (GenBank: CY147535.1) was cloned into the pEXPR-IBA 103 vector (IBA Lifesciences, Göttingen, Germany), and the plasmid DNA was transfected into ExpiCHO cells. Five days later, the cells were harvested and lysed by sonication (30% amplitude, 10 s on/10 s off, total 10 min). After filtration with a 0.45 μm filter (Corning), the lysate supernatant was bound to Strep-Tactin XT resin (IBA Lifesciences) using a gravity flow column. After washing, the bound protein was collected by treating the column with an elution buffer (IBA Lifesciences). The endotoxin level in the purified M1 was measured using the endotoxin test kit (Thermo Fisher Scientific, Waltham, Massachusetts, USA). The protein was stored at –80 °C until further use.

### Virus

The influenza A/Puerto Rico/8/1934 (PR8) virus was cultivated in the allantoic cavity of embryonated chicken eggs. Viruses were titrated by calculating the 50% egg infectious dose (EID_50_) and 50% tissue culture infectious dose (TCID_50_) and stored at –80 °C until use.

### Anti-M1 polyserum and anti-M1 antibody

The M1-coding DNA fragment was cloned into the pGX-10 plasmid [48], which was used as the DNA vaccine vector in this study. Mice (BALB/c) were intramuscularly immunized twice at 3-week intervals with the plasmid DNA (10 μg) using electroporation. Serum was obtained 2 weeks after the final vaccination. Anti-M1 IgG was purified from the serum of mock- and M1-vaccinated mice using an IgG antibody purification kit (Abcam, Cambridge, UK) according to the manufacturer’s protocol. Serum and purified antibodies were stored at –20 °C.

### Preparation of lung samples (whole lung cells, BAL fluid, and BAL cells)

Total lung cells and BAL fluid samples were obtained as described previously [8]. Briefly, lung tissue was minced and incubated in 1.5 ml RPMI 1640 media containing collagenase D (150 unit/ml, Gibco), DNase I (50 μg/ml, Merck), 10% FBS, and 1× antibiotics. After enzymatic digestion, cells were isolated from the tissue using a strainer (SPL, Pyeongtaek, Korea). For BAL fluid preparation, a catheter was inserted in the trachea of anesthetized mice and 1 ml of cold PBS containing protease inhibitors (Merck) was instilled into the lungs. After gentle aspiration, BAL fluid and cells were separated by centrifugation (4000 rpm, 5 min, 4 °C).

### Experimental schedule

Mice (C57BL/6) were treated intranasally with recombinant M1 protein (rM1), after which, BAL fluid, BAL cells and lung cells were obtained at each indicated time point for enzyme-linked immunosorbent assay (ELISA), western blotting, and flow cytometry analysis. Mice (C57BL/6) infected with PR8 influenza virus (32 PFU) were intranasally treated with PBS or M1 immediately, 3 days pre- or post-infection. Influenza virus-infected mice were intraperitoneally administered with 200 μl of naïve or anti-M1 serum or 15–200 μg of anti-M1 purified IgG antibody daily for 3 days. The change in bodyweight and survival rate of the mice were monitored for 14 days post infection.

### Western blot

Cells were lysed in CETi lysis buffer (TransLab, Daejeon, Korea) and the lysate was separated by sodium dodecyl-sulfate polyacrylamide gel electrophoresis (SDS-PAGE). After transferring the proteins onto polyvinylidene fluoride membranes (Merck), the membranes were blocked and incubated with primary antibodies overnight at 4 °C. After washing, horseradish peroxidase (HRP)-conjugated secondary antibodies were added. Bound antibodies were visualized using Immobilon Forte Western HRP substrate (Merck) and imaged using EZ-Capture II (ATTO Corporation, Tokyo, Japan). The following antibodies were used: anti-phospho-SAPK/JNK (Cat# 9255), anti-SAPK/JNK (Cat# 9252), anti-phospho-p44/42 MAPK (Cat# 4370), anti-p44/42 (Cat# 4695), anti-phospho-p38 MAPK (Cat# 4511), anti-p38 MAPK (Cat# 54470), anti-phospho-IκB (Cat# 2859), anti-IκB-alpha (Cat# 4812), anti-phospho-NF-κB p65 (Cat# 3033), anti-COX1 (Cat# 9896), anti-COX2 (Cat# 12282), anti-iNOS (Cat# 13120), anti-Bim (Cat# 2933), anti-phospho-Bad (Cat# 5284), anti-Bak (Cat# 12105), anti-caspase-3 (Cat# 9665), anti-caspase-6 (Cat# 9762), anti-caspase-7 (Cat# 8438), anti-caspase-9 (Cat# 9508), HRP-linked anti-mouse IgG (Cat# 7076), HRP-linked anti-rabbit IgG (Cat# 7074),(all from CST, Danvers, Massachusetts, USA), and anti-Strep-tag II (Abcam, Cat# ab76949).

### IP

For IP, BMDMs were lysed in CETi lysis buffer for 10 min on ice. Lysates were centrifuged and the supernatants were transferred to fresh tubes containing antibody coated Dynabeads as per the manufacturer’s instructions (Invitrogen), followed by western blot analysis. The following antibodies were used: anti-TLR4 (Santa Cruz Biotechnology, Dallas, Texas, USA; and Thermo Fisher Scientific, Cat# sc-293072) and anti-influenza M1 (Sino biological, Beijing, China, Cat# 40010-RP01).

### ELISA, LDH, and ROS assay

BMDM, MLE-12, and BAL cell culture supernatants were collected after treatment with M1 or LPS for 20 h for ELISA and after 0–48 h for LDH and ROS assays. BAL fluid samples were obtained from the mice, and cytokines were measured using IL-6, CXCL1, CXCL-10, CCL2, CCL5 ELISA kits (all from R&D Systems, Minneapolis, Minnesota, USA). LDH and ROS were detected using the CyQUANT™ LDH cytotoxicity assay (Invitrogen) and in vitro ROS/RNS assay kits (Cell Biolabs Inc. San Diego, CA, US), respectively, according to the manufacturers’ protocols.

### RNA sequencing and analysis

#### (1) RNA isolation

BMDM cells were treated with PBS, M1, NP, or LPS for 6 h, after which total RNA was isolated using Trizol reagent (Invitrogen Corp., Carlsbad, CA, USA). RNA purity and integrity were evaluated using a ND-2000 Spectrophotometer (NanoDrop, Wilmington, USA) and Agilent 2100 Bioanalyzer (Agilent Technologies, Palo Alto, USA), respectively.

#### (2) Affymetrix whole-transcript expression array

The Affymetrix Whole-transcript Expression array was performed using the GeneChip Whole Transcript PLUS reagent Kit (Thermo Fisher Scientific) per the manufacturer’s protocol. cDNA was synthesized using the GeneChip WT (Whole Transcript) Amplification kit as described by the manufacturer. The sense cDNA was then fragmented and biotin-labeled with terminal deoxynucleotidyl transferase (TdT) using the GeneChip WT Terminal labeling kit. Approximately 5.5 μg of labeled DNA target was hybridized to the Affymetrix GeneChip Mouse ST 2.0 Array at 45 °C for 16 h. Hybridized arrays were washed and stained on a GeneChip Fluidics Station 450 and scanned on a GCS3000 Scanner (Affymetrix). Signal values were computed using the Affymetrix® GeneChip™ Command Console software.

#### (3) Data analysis

Expression data were generated by Transcriptome Analysis console 4.0.1. For the normalization, robust multi-average (RMA) algorithm implemented in Transcriptome Analysis Console software was used. Data mining and graphic visualization were performed using ExDEGA (Ebiogen Inc., Korea). To define differentially expressed genes (DEGs), adjusted |log2fold-change (FC)| ≥ 1.5 and *P* < 0.05 were selected as cut-off values.

### TLR reporter cell line assay

The TLR reporter cell lines (Invivogen) were cultured in DMEM containing 10% FBS and 1× antibiotics. When the HEK-Blue™ TLR4 reporter cells reached 80% confluency, they were harvested using a scraper and resuspended in HEK-Blue™ Detection media (InvivoGen). The resuspended cells (5 × 10^4^ cells/well; 180 μl) were then incubated with 20 μl of each stimulant in a 96-well plate. After 20 h of culture, the optical density at 630 nm (OD_630_) was measured.

### Flow cytometry

Single lung cell suspensions were blocked using anti-mouse CD16/CD32 (mouse BD Fc Block™; BD Biosciences) at room temperature for 15 min before staining. The surface antigens were stained with the indicated conjugated antibodies at 4 °C for 15 min. The following antibodies were used: APC anti-CD4 (Thermo Fisher Scientific, Cat# 17-0042-82), PerCP-eFluor 710 anti-CD3e (Thermo Fisher Scientific, Cat# 46-0033-82), PE/Cyanine7 anti-CD45R (Thermo Fisher Scientific, Cat# 25-0452-82), PE anti-Siglec-F (Thermo Fisher Scientific, Cat# 552126), PerCD-eFluor 710 anti-Ly6G (Thermo Fisher Scientific, Cat# 46-9668-82), Alexa Fluor 700 anti-MHC Class II I-A/I-E (Thermo Fisher Scientific, Cat# 56-5321-80), and eFluor 450 anti-F4/80 (Thermo Fisher Scientific, Cat# 48-4801-82); APC/cyanine7 anti-CD45 (BioLegend, San Diego, CA, USA, Cat# 103116); APC anti-CD11b (BioLegend, Cat# 101212) and PE anti-CD49b (BioLegend, Cat# 103506); and FITC anti-NK1.1 (BD Biosciences, Cat# 553164). Stained cells were analyzed using a Gallios flow cytometer (Beckman Coulter, Brea, CA, US) with FlowJo™ software (Becton, Dickinson and Company, New Jersey, USA).

### Statistical analysis

Statistical differences among groups were assessed using a two-tailed Student’s t-test and a log-rank test with Prism software (GraphPad Software, USA). Mice were randomly assigned to experimental groups without blinding method for injections. No animal exclusion criteria were applied, and the variance was comparable among the groups that were statistically compared. Values with *p* < 0.05 were considered to be statistically significant.

## Supplementary information

Supplementary Figure 1-7

Checklist

Original Data File

## Data Availability

All datasets generated and analysed during this study are included in this published article and its Supplementary Information files. Additional data are available from the corresponding author on reasonable request.

## References

[1] Julkunen I, Melen K, Nyqvist M, Pirhonen J, Sareneva T, Matikainen S (2000). Inflammatory responses in influenza A virus infection. Vaccine.

[2] Lee SM, Cheung CY, Nicholls JM, Hui KP, Leung CY, Uiprasertkul M (2008). Hyperinduction of cyclooxygenase-2-mediated proinflammatory cascade: a mechanism for the pathogenesis of avian influenza H5N1 infection. J Infect Dis.

[3] Wareing MD, Lyon AB, Lu B, Gerard C, Sarawar SR (2004). Chemokine expression during the development and resolution of a pulmonary leukocyte response to influenza A virus infection in mice. J Leukoc Biol.

[4] Chen W, Calvo PA, Malide D, Gibbs J, Schubert U, Bacik I (2001). A novel influenza A virus mitochondrial protein that induces cell death. Nat Med.

[5] Chang P, Kuchipudi SV, Mellits KH, Sebastian S, James J, Liu J (2015). Early apoptosis of porcine alveolar macrophages limits avian influenza virus replication and pro-inflammatory dysregulation. Sci Rep.

[6] Lam WY, Tang JW, Yeung AC, Chiu LC, Sung JJ, Chan PK (2008). Avian influenza virus A/HK/483/97(H5N1) NS1 protein induces apoptosis in human airway epithelial cells. J Virol.

[7] Hartmann BM, Albrecht RA, Zaslavsky E, Nudelman G, Pincas H, Marjanovic N (2017). Pandemic H1N1 influenza A viruses suppress immunogenic RIPK3-driven dendritic cell death. Nat Commun.

[8] Kim C-U, Jeong Y-J, Lee P, Lee M-S, Park J-H, Kim Y-S (2022). Extracellular nucleoprotein exacerbates influenza virus pathogenesis by activating Toll-like receptor 4 and the NLRP3 inflammasome. Cell Mol Immunolog.

[9] Peukes J, Xiong X, Erlendsson S, Qu K, Wan W, Calder LJ (2020). The native structure of the assembled matrix protein 1 of influenza A virus. Nature.

[10] Halder UC, Bagchi P, Chattopadhyay S, Dutta D, Chawla-Sarkar M (2011). Cell death regulation during influenza A virus infection by matrix (M1) protein: a model of viral control over the cellular survival pathway. Cell Death Dis.

[11] Zhirnov OP, Ksenofontov AL, Kuzmina SG, Klenk HD (2002). Interaction of influenza A virus M1 matrix protein with caspases. Biochemistry (Mosc).

[12] Imai Y, Kuba K, Neely GG, Yaghubian-Malhami R, Perkmann T, van Loo G (2008). Identification of oxidative stress and Toll-like receptor 4 signaling as a key pathway of acute lung injury. Cell.

[13] Pone EJ, Hernandez-Davies JE, Jan S, Silzel E, Felgner PL, Davies DH (2022). Multimericity amplifies the synergy of BCR and TLR4 for B cell activation and antibody class switching. Front Immunol.

[14] Selzer L, Su Z, Pintilie GD, Chiu W, Kirkegaard K (2020). Full-length three-dimensional structure of the influenza A virus M1 protein and its organization into a matrix layer. PLoS Biol.

[15] Zhao H, Ekstrom M, Garoff H (1998). The M1 and NP proteins of influenza A virus form homo- but not heterooligomeric complexes when coexpressed in BHK-21 cells. J Gen Virol.

[16] Chen H, Ning X, Jiang Z (2017). Caspases control antiviral innate immunity. Cell Mol Immunol.

[17] Redza-Dutordoir M, Averill-Bates DA (2016). Activation of apoptosis signalling pathways by reactive oxygen species. Biochim Biophys Acta.

[18] Herold S, Steinmueller M, von Wulffen W, Cakarova L, Pinto R, Pleschka S (2008). Lung epithelial apoptosis in influenza virus pneumonia: the role of macrophage-expressed TNF-related apoptosis-inducing ligand. J Exp Med.

[19] Hogner K, Wolff T, Pleschka S, Plog S, Gruber AD, Kalinke U (2013). Macrophage-expressed IFN-beta contributes to apoptotic alveolar epithelial cell injury in severe influenza virus pneumonia. PLoS Pathog.

[20] Rodrigue-Gervais IG, Labbé K, Dagenais M, Dupaul-Chicoine J, Champagne C, Morizot A (2014). Cellular inhibitor of apoptosis protein cIAP2 protects against pulmonary tissue necrosis during influenza virus infection to promote host survival. Cell Host Microbe.

[21] Mosavi SZ, Shahsavandi S, Ebrahimi MM, Hatami AR, Sadeghi K, Shahivandi H (2015). Necrotic response to low pathogenic H9N2 influenza virus in chicken hepatoma cells. Jundishapur J Microbiol.

[22] Arndt U, Wennemuth G, Barth P, Nain M, Al-Abed Y, Meinhardt A (2002). Release of macrophage migration inhibitory factor and CXCL8/interleukin-8 from lung epithelial cells rendered necrotic by influenza A virus infection. J Virol.

[23] Balachandran S, Rall GF (2020). Benefits and perils of necroptosis in influenza virus infection. J Virol.

[24] Vanden Berghe T, Vanlangenakker N, Parthoens E, Deckers W, Devos M, Festjens N (2010). Necroptosis, necrosis and secondary necrosis converge on similar cellular disintegration features. Cell Death Differ.

[25] Hofer CT, Di Lella S, Dahmani I, Jungnick N, Bordag N, Bobone S (2019). Structural determinants of the interaction between influenza A virus matrix protein M1 and lipid membranes. Biochim Biophys Acta Biomembr.

[26] Heer AK, Shamshiev A, Donda A, Uematsu S, Akira S, Kopf M (2007). TLR signaling fine-tunes anti-influenza B cell responses without regulating effector T cell responses. J Immunol.

[27] Koyama S, Aoshi T, Tanimoto T, Kumagai Y, Kobiyama K, Tougan T (2010). Plasmacytoid dendritic cells delineate immunogenicity of influenza vaccine subtypes. Sci Transl Med.

[28] Koyama S, Ishii KJ, Kumar H, Tanimoto T, Coban C, Uematsu S (2007). Differential role of TLR- and RLR-signaling in the immune responses to influenza A virus infection and vaccination. J Immunol.

[29] Lopez CB, Moltedo B, Alexopoulou L, Bonifaz L, Flavell RA, Moran TM (2004). TLR-independent induction of dendritic cell maturation and adaptive immunity by negative-strand RNA viruses. J Immunol.

[30] Allen IC, Scull MA, Moore CB, Holl EK, McElvania-TeKippe E, Taxman DJ (2009). The NLRP3 inflammasome mediates in vivo innate immunity to influenza A virus through recognition of viral RNA. Immunity.

[31] Ichinohe T, Lee HK, Ogura Y, Flavell R, Iwasaki A (2009). Inflammasome recognition of influenza virus is essential for adaptive immune responses. J Exp Med.

[32] Thomas PG, Dash P, Aldridge JR, Ellebedy AH, Reynolds C, Funk AJ (2009). The intracellular sensor NLRP3 mediates key innate and healing responses to influenza A virus via the regulation of caspase-1. Immunity.

[33] Uiprasertkul M, Kitphati R, Puthavathana P, Kriwong R, Kongchanagul A, Ungchusak K (2007). Apoptosis and pathogenesis of avian influenza A (H5N1) virus in humans. Emerg Infect Dis.

[34] Chen KK, Minakuchi M, Wuputra K, Ku CC, Pan JB, Kuo KK (2020). Redox control in the pathophysiology of influenza virus infection. BMC Microbiol.

[35] Mantegazza AR, Savina A, Vermeulen M, Perez L, Geffner J, Hermine O (2008). NADPH oxidase controls phagosomal pH and antigen cross-presentation in human dendritic cells. Blood.

[36] Matsue H, Edelbaum D, Shalhevet D, Mizumoto N, Yang C, Mummert ME (2003). Generation and function of reactive oxygen species in dendritic cells during antigen presentation. J Immunol.

[37] Oberkampf M, Guillerey C, Mouries J, Rosenbaum P, Fayolle C, Bobard A (2018). Mitochondrial reactive oxygen species regulate the induction of CD8(+) T cells by plasmacytoid dendritic cells. Nat Commun.

[38] Akira S, Uematsu S, Takeuchi O (2006). Pathogen recognition and innate immunity. Cell.

[39] Molteni M, Gemma S, Rossetti C (2016). The Role of Toll-Like Receptor 4 in Infectious and Noninfectious Inflammation. Mediators Inflamm.

[40] Mata-Haro V, Cekic C, Martin M, Chilton PM, Casella CR, Mitchell TC (2007). The vaccine adjuvant monophosphoryl lipid A as a TRIF-biased agonist of TLR4. Science.

[41] Ohto U, Fukase K, Miyake K, Satow Y (2007). Crystal structures of human MD-2 and its complex with antiendotoxic lipid IVa. Science.

[42] Cui W, Joshi NS, Liu Y, Meng H, Kleinstein SH, Kaech SM (2014). TLR4 ligands lipopolysaccharide and monophosphoryl lipid a differentially regulate effector and memory CD8+ T Cell differentiation. J Immunol.

[43] Luan L, Patil NK, Guo Y, Hernandez A, Bohannon JK, Fensterheim BA (2017). Comparative transcriptome profiles of human blood in response to the toll-like receptor 4 ligands lipopolysaccharide and monophosphoryl lipid A. Sci Rep.

[44] Padilla-Quirarte HO, Lopez-Guerrero DV, Gutierrez-Xicotencatl L, Esquivel-Guadarrama F (2019). Protective antibodies against influenza proteins. Front Immunol.

[45] Vanderven HA, Ana-Sosa-Batiz F, Jegaskanda S, Rockman S, Laurie K, Barr I (2016). What lies beneath: antibody dependent natural killer cell activation by antibodies to internal influenza virus proteins. EBioMedicine.

[46] Carragher DM, Kaminski DA, Moquin A, Hartson L, Randall TD (2008). A novel role for non-neutralizing antibodies against nucleoprotein in facilitating resistance to influenza virus. J Immunol.

[47] LaMere MW, Lam HT, Moquin A, Haynes L, Lund FE, Randall TD (2011). Contributions of antinucleoprotein IgG to heterosubtypic immunity against influenza virus. J Immunol.

[48] Ha SJ, Jeon BY, Kim SC, Kim DJ, Song MK, Sung YC (2003). Therapeutic effect of DNA vaccines combined with chemotherapy in a latent infection model after aerosol infection of mice with mycobacterium tuberculosis. Gene Ther.

