# Supplementary material for: Influenza viral matrix 1 protein aggravates viral pathogenicity by inducing TLR4-mediated reactive oxygen species production and apoptotic cell death
Source: Cell Death Dis. 2023 Mar 30;14(3):228. doi: 10.1038/s41419-023-05749-5 (PMC10060384; doi:10.1038/s41419-023-05749-5)
Supplement: Supplementary file 1 — Supplementary Figure 1-7 [file 41419_2023_5749_MOESM1_ESM.pptx]

## Slide 1
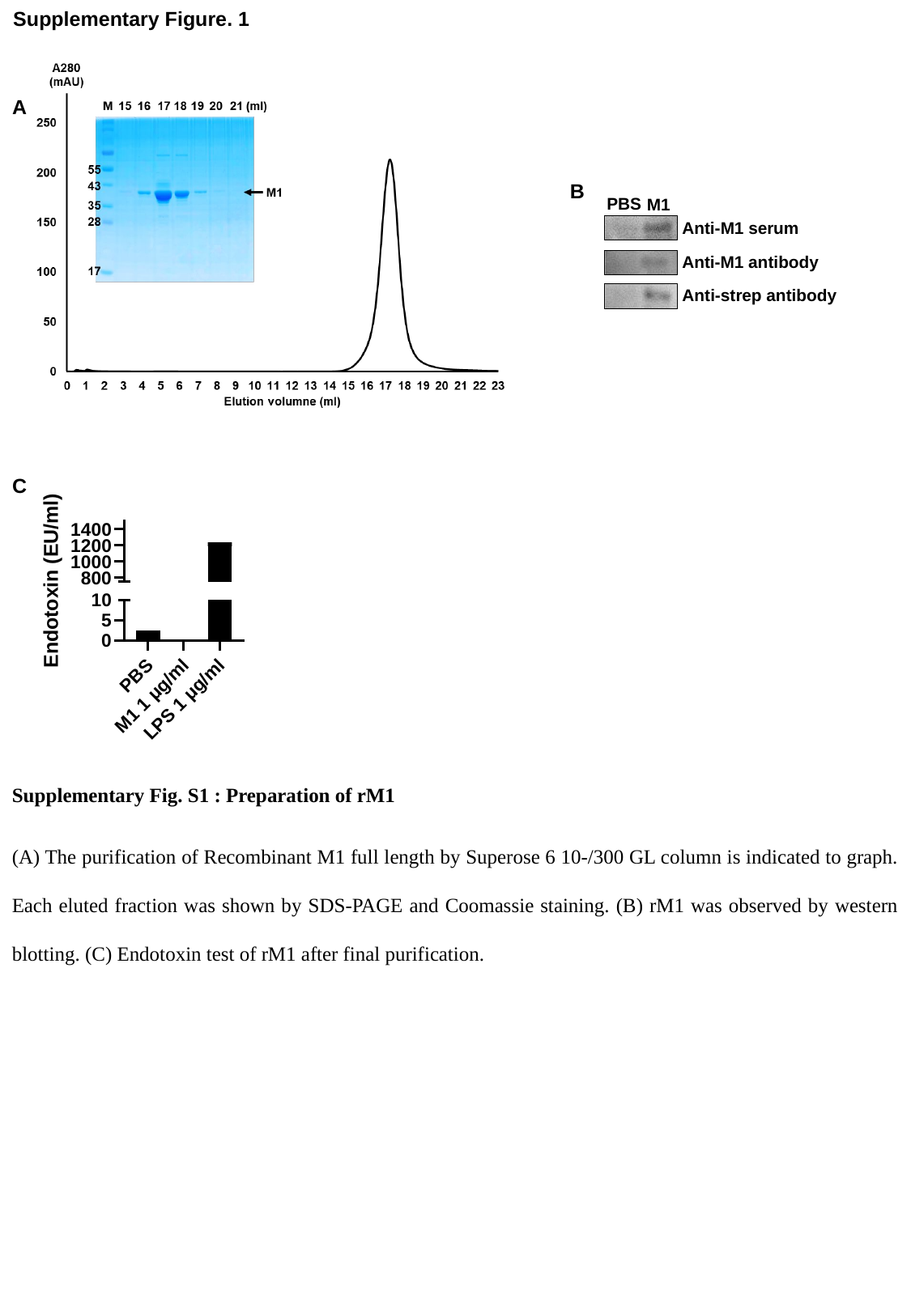

Supplementary Figure. 1
A
B
PBS
M1
Anti-M1 serum
Anti-M1 antibody
Anti-strep antibody
C
Supplementary Fig. S1 : Preparation of rM1
(A) The purification of Recombinant M1 full length by Superose 6 10-/300 GL column is indicated to graph. Each eluted fraction was shown by SDS-PAGE and Coomassie staining. (B) rM1 was observed by western blotting. (C) Endotoxin test of rM1 after final purification.

## Slide 2
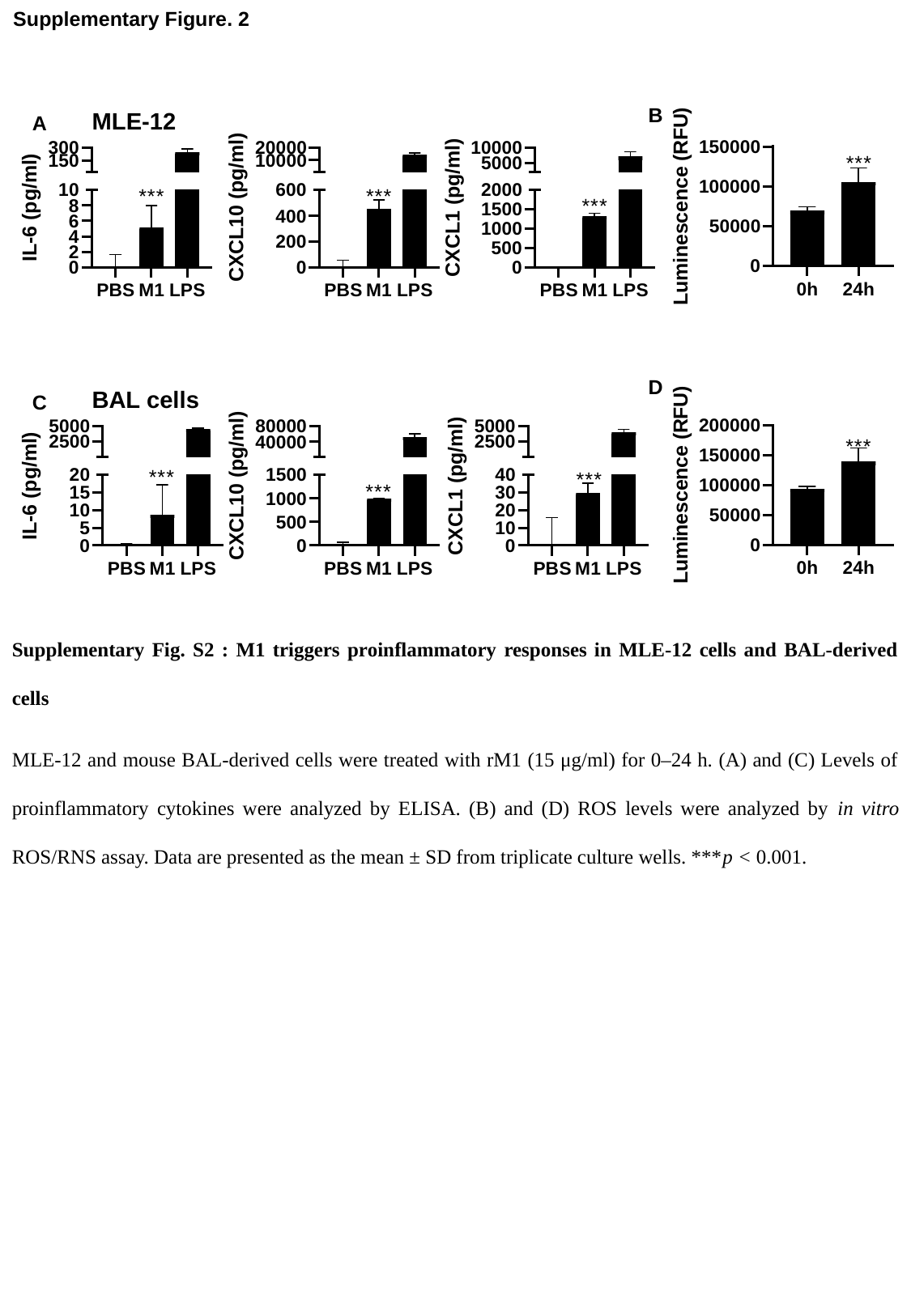

Supplementary Figure. 2
B
MLE-12
A
D
BAL cells
C
Supplementary Fig. S2 : M1 triggers proinflammatory responses in MLE-12 cells and BAL-derived cells
MLE-12 and mouse BAL-derived cells were treated with rM1 (15 μg/ml) for 0–24 h. (A) and (C) Levels of proinflammatory cytokines were analyzed by ELISA. (B) and (D) ROS levels were analyzed by in vitro ROS/RNS assay. Data are presented as the mean ± SD from triplicate culture wells. ***p < 0.001.

## Slide 3
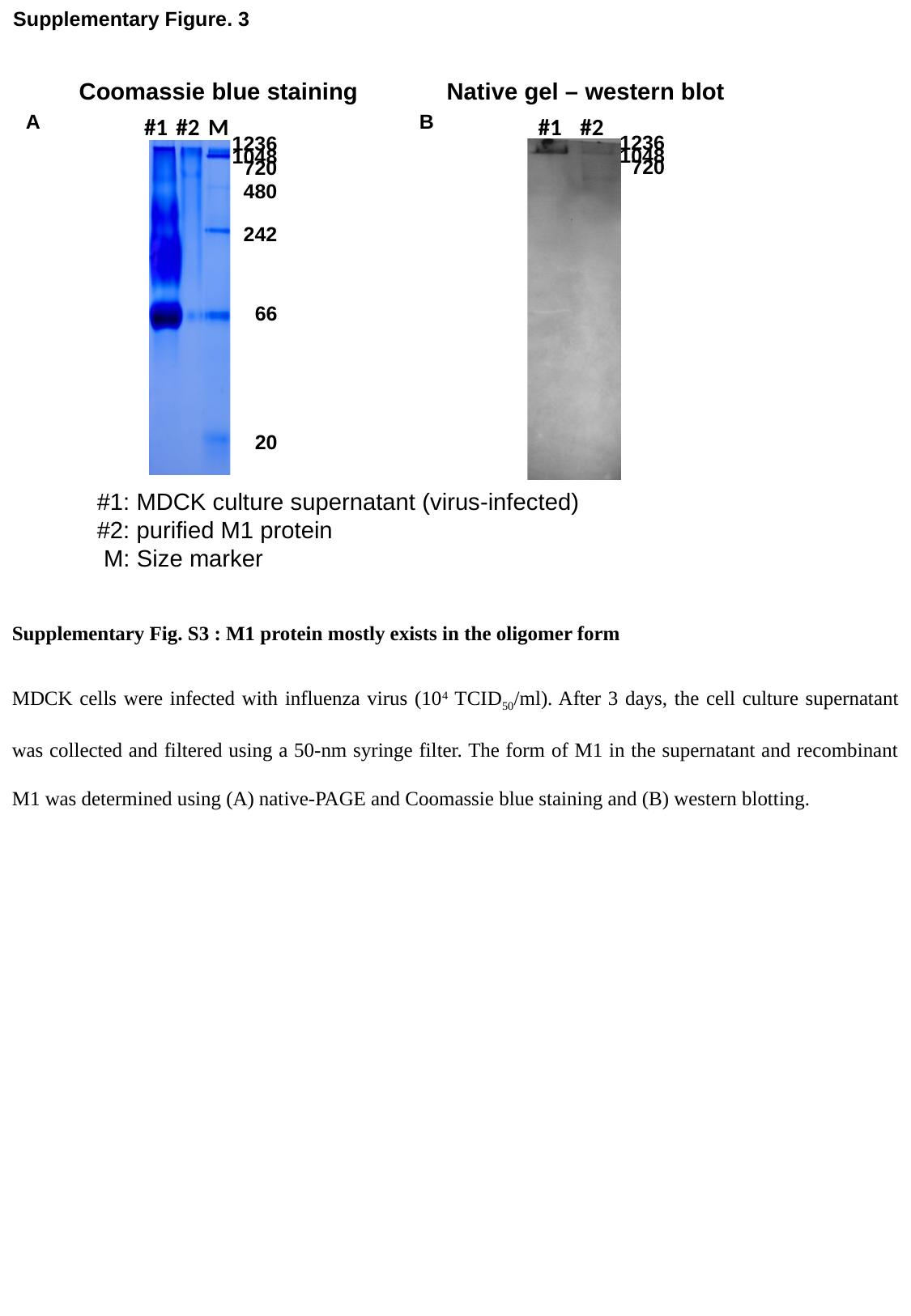

Supplementary Figure. 3
Coomassie blue staining
Native gel – western blot
A
B
#1
#2
M
#1
#2
1236
1236
1048
1048
720
720
480
242
66
20
#1: MDCK culture supernatant (virus-infected)
#2: purified M1 protein
 M: Size marker
Supplementary Fig. S3 : M1 protein mostly exists in the oligomer form
MDCK cells were infected with influenza virus (104 TCID50/ml). After 3 days, the cell culture supernatant was collected and filtered using a 50-nm syringe filter. The form of M1 in the supernatant and recombinant M1 was determined using (A) native-PAGE and Coomassie blue staining and (B) western blotting.

## Slide 4
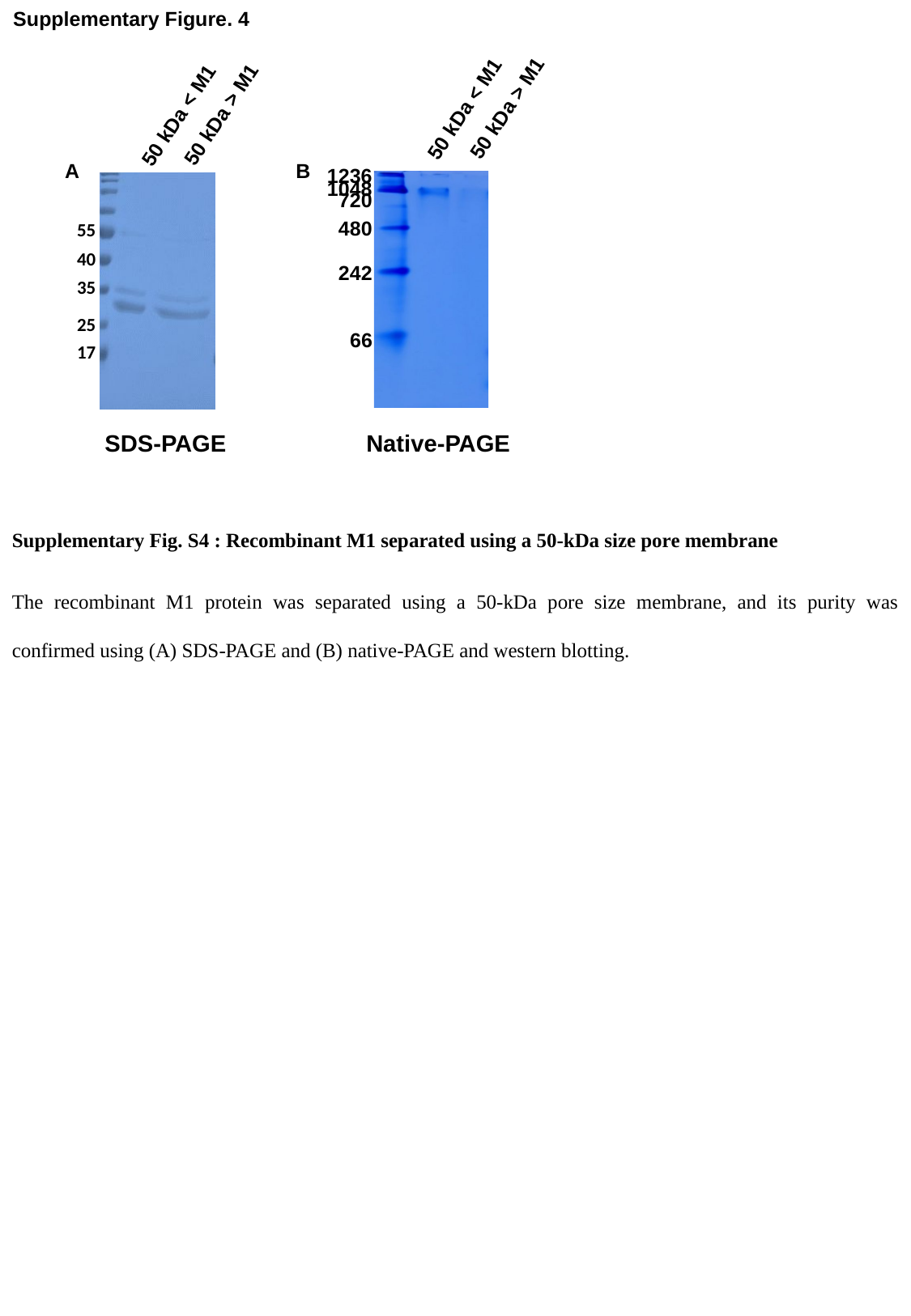

Supplementary Figure. 4
50 kDa > M1
50 kDa < M1
1236
1048
720
480
242
66
Native-PAGE
50 kDa > M1
50 kDa < M1
55
40
35
25
17
SDS-PAGE
A
B
Supplementary Fig. S4 : Recombinant M1 separated using a 50-kDa size pore membrane
The recombinant M1 protein was separated using a 50-kDa pore size membrane, and its purity was confirmed using (A) SDS-PAGE and (B) native-PAGE and western blotting.

## Slide 5
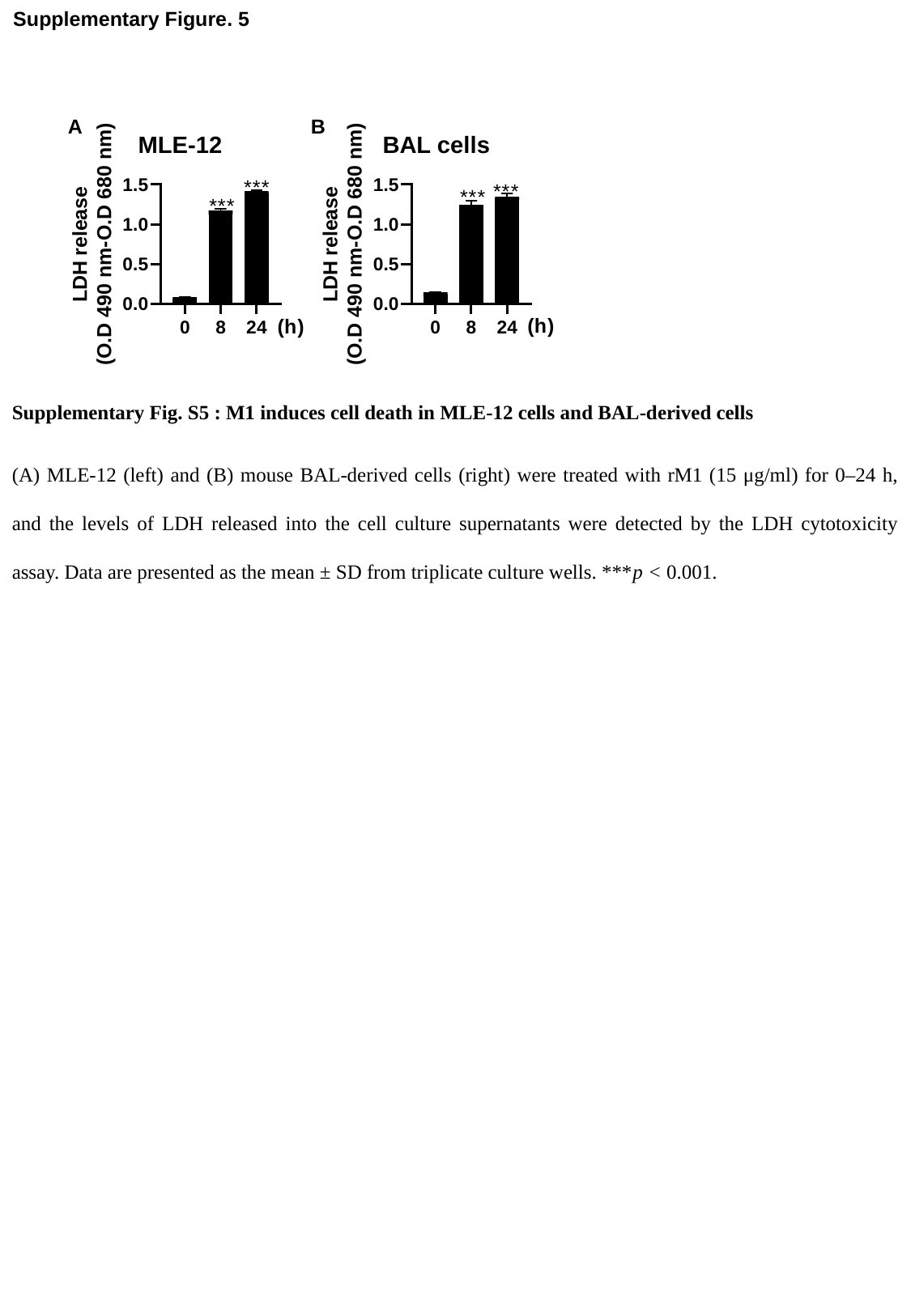

Supplementary Figure. 5
A
B
MLE-12
BAL cells
Supplementary Fig. S5 : M1 induces cell death in MLE-12 cells and BAL-derived cells
(A) MLE-12 (left) and (B) mouse BAL-derived cells (right) were treated with rM1 (15 μg/ml) for 0–24 h, and the levels of LDH released into the cell culture supernatants were detected by the LDH cytotoxicity assay. Data are presented as the mean ± SD from triplicate culture wells. ***p < 0.001.

## Slide 6
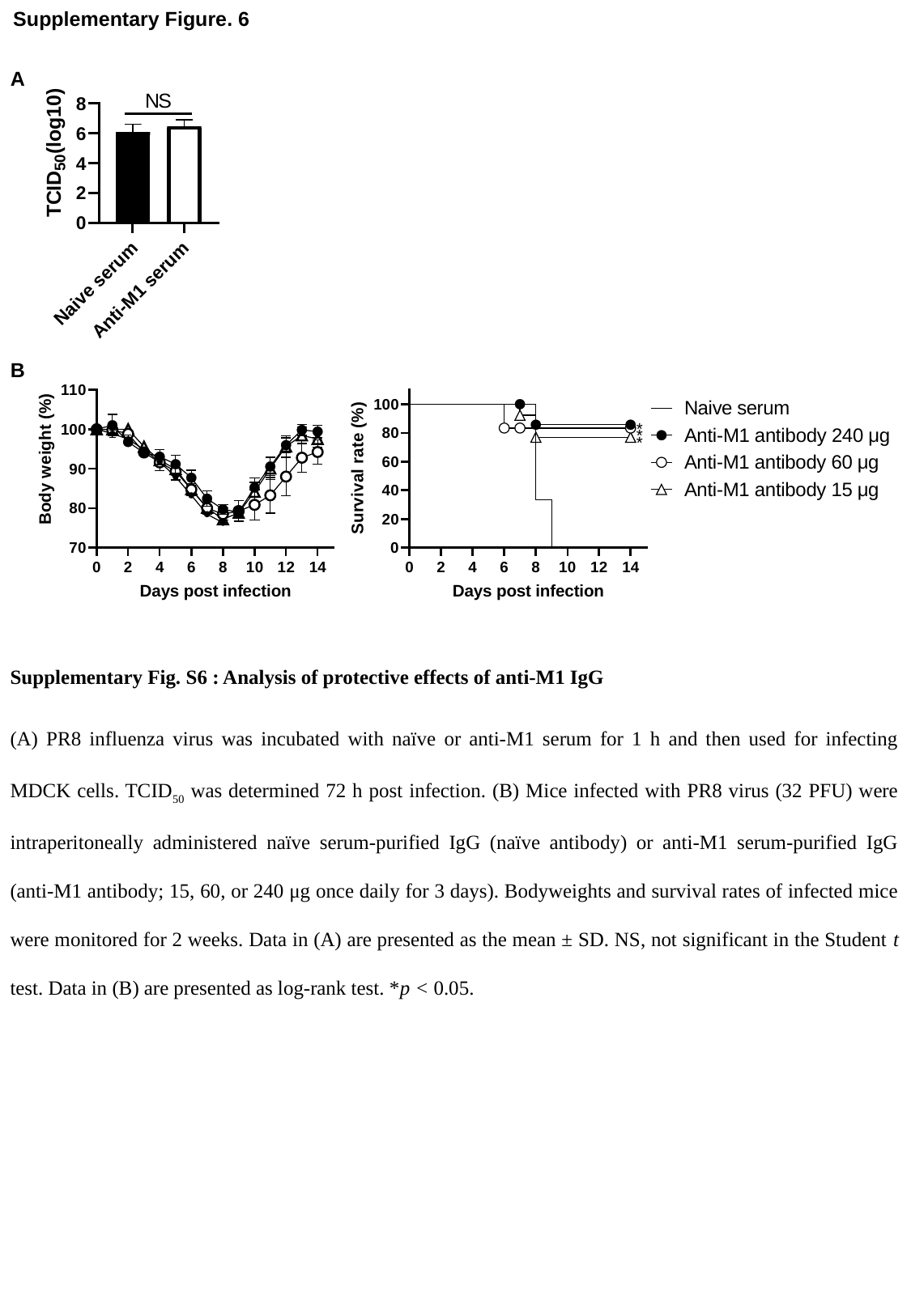

Supplementary Figure. 6
A
B
Supplementary Fig. S6 : Analysis of protective effects of anti-M1 IgG
(A) PR8 influenza virus was incubated with naïve or anti-M1 serum for 1 h and then used for infecting MDCK cells. TCID50 was determined 72 h post infection. (B) Mice infected with PR8 virus (32 PFU) were intraperitoneally administered naïve serum-purified IgG (naïve antibody) or anti-M1 serum-purified IgG (anti-M1 antibody; 15, 60, or 240 μg once daily for 3 days). Bodyweights and survival rates of infected mice were monitored for 2 weeks. Data in (A) are presented as the mean ± SD. NS, not significant in the Student t test. Data in (B) are presented as log-rank test. *p < 0.05.

## Slide 7
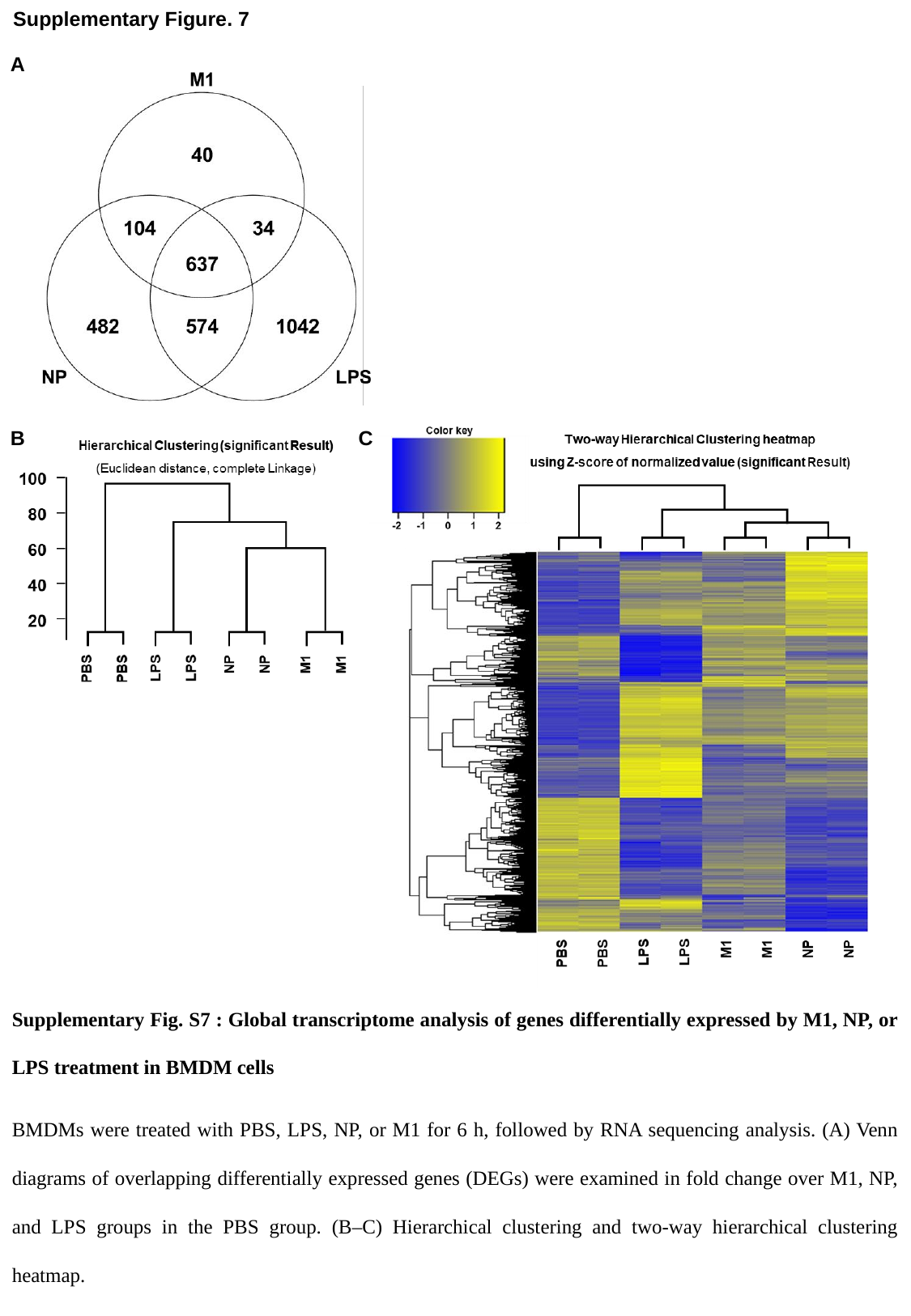

Supplementary Figure. 7
A
B
C
Supplementary Fig. S7 : Global transcriptome analysis of genes differentially expressed by M1, NP, or LPS treatment in BMDM cells
BMDMs were treated with PBS, LPS, NP, or M1 for 6 h, followed by RNA sequencing analysis. (A) Venn diagrams of overlapping differentially expressed genes (DEGs) were examined in fold change over M1, NP, and LPS groups in the PBS group. (B–C) Hierarchical clustering and two-way hierarchical clustering heatmap.
