# Supplementary material for: Influenza viral matrix 1 protein aggravates viral pathogenicity by inducing TLR4-mediated reactive oxygen species production and apoptotic cell death
Source: Cell Death Dis. 2023 Mar 30;14(3):228. doi: 10.1038/s41419-023-05749-5 (PMC10060384; doi:10.1038/s41419-023-05749-5)
Supplement: Supplementary file 3 — Original Data File [file 41419_2023_5749_MOESM3_ESM.pptx]

## Slide 1
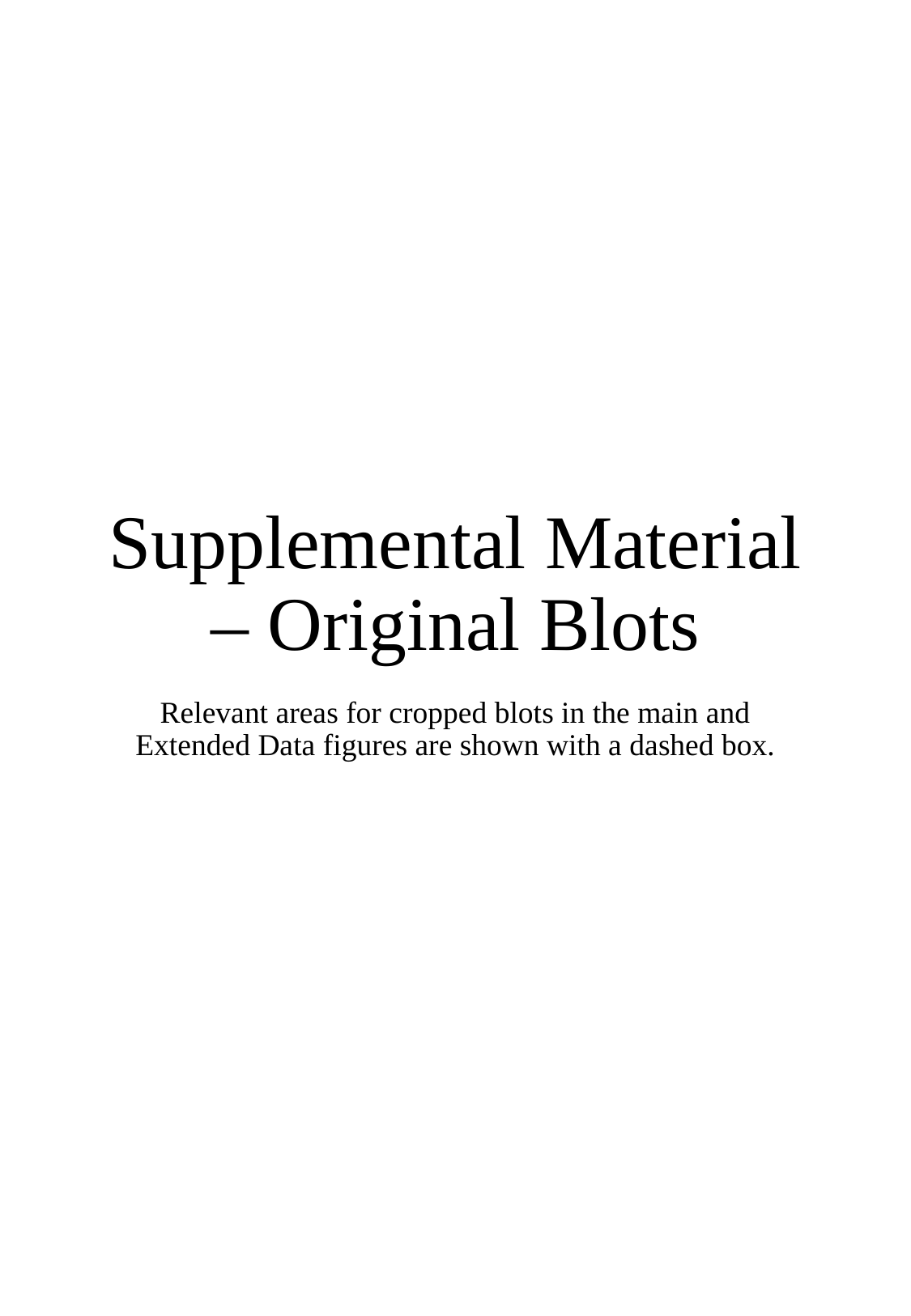

# Supplemental Material – Original Blots
Relevant areas for cropped blots in the main and Extended Data figures are shown with a dashed box.

## Slide 2
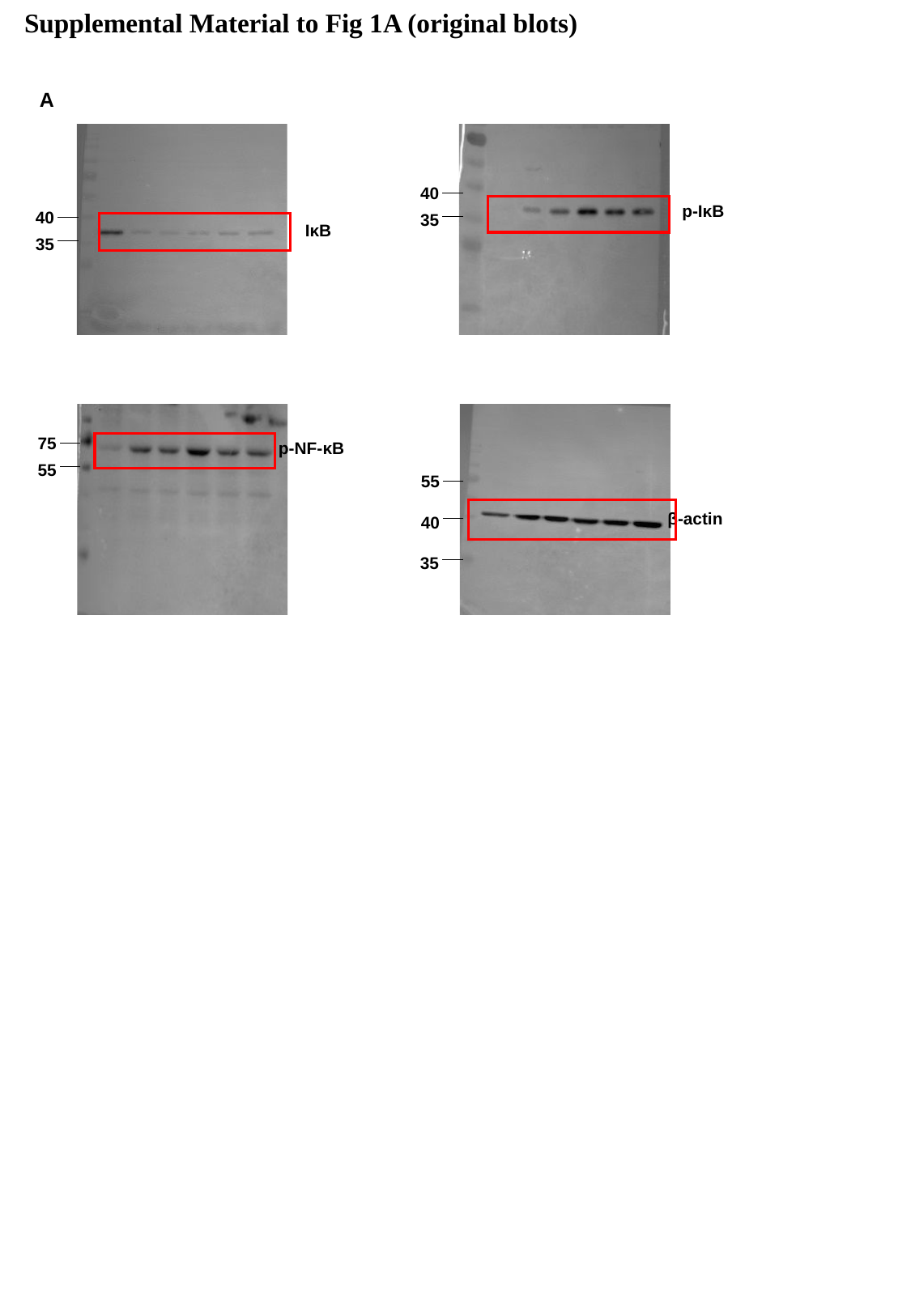

Supplemental Material to Fig 1A (original blots)
A
IκB
p-IκB
40
40
35
35
p-NF-κB
β-actin
75
55
55
40
35

## Slide 3
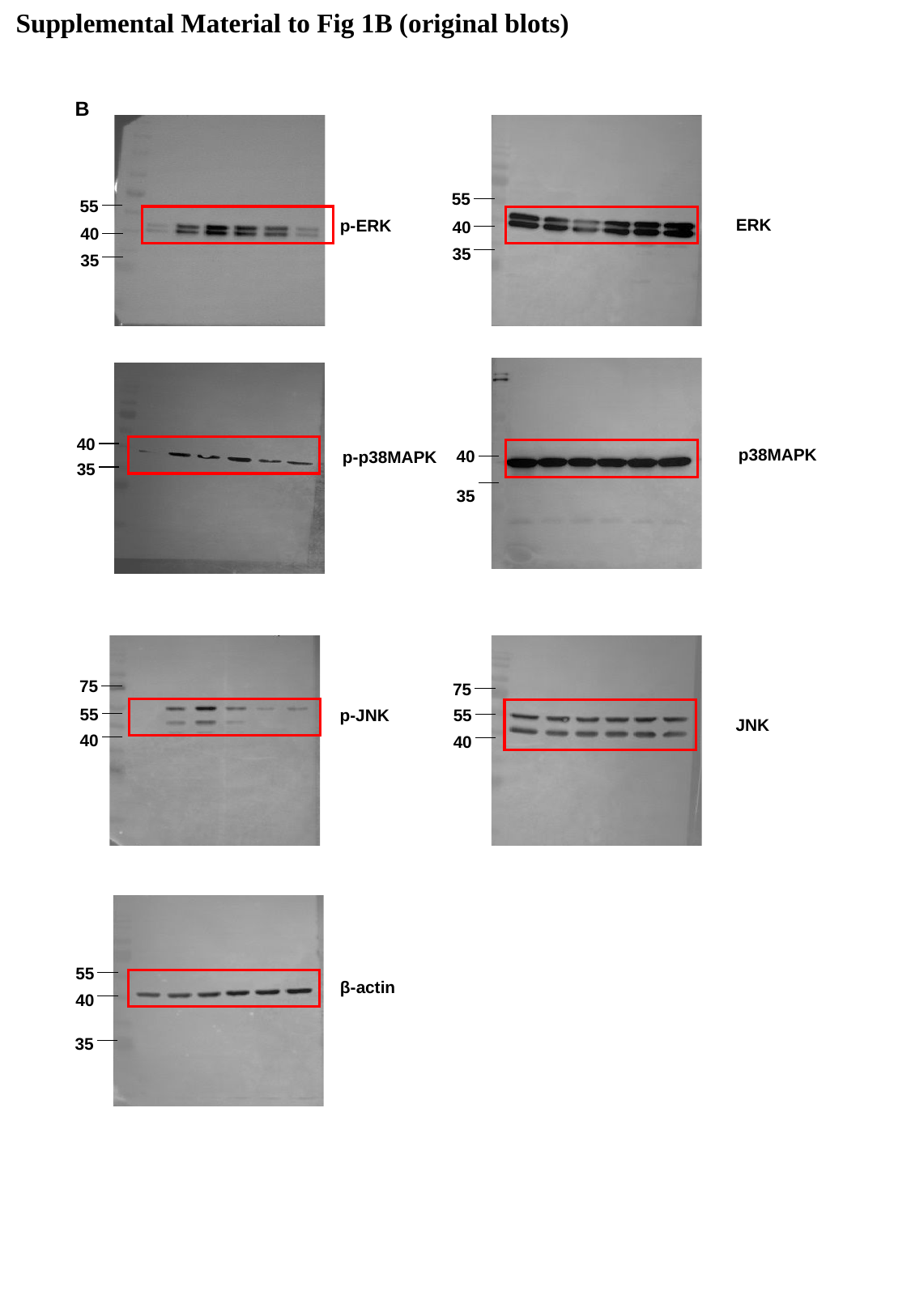

Supplemental Material to Fig 1B (original blots)
B
55
55
ERK
p-ERK
40
40
35
35
40
p38MAPK
40
p-p38MAPK
35
35
75
75
55
55
p-JNK
JNK
40
40
55
β-actin
40
35

## Slide 4
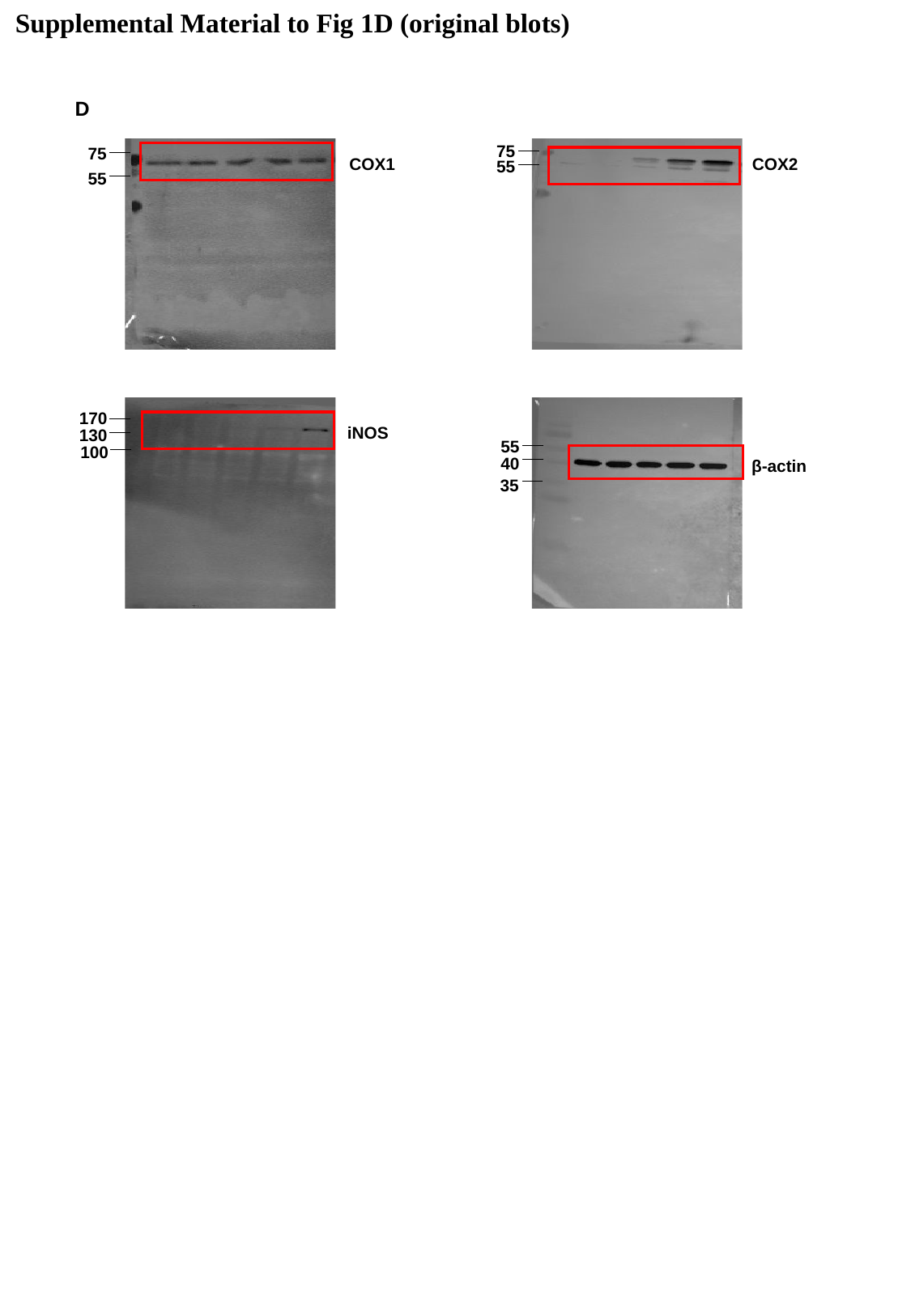

Supplemental Material to Fig 1D (original blots)
D
75
75
COX2
COX1
55
55
170
iNOS
130
55
100
40
β-actin
35

## Slide 5
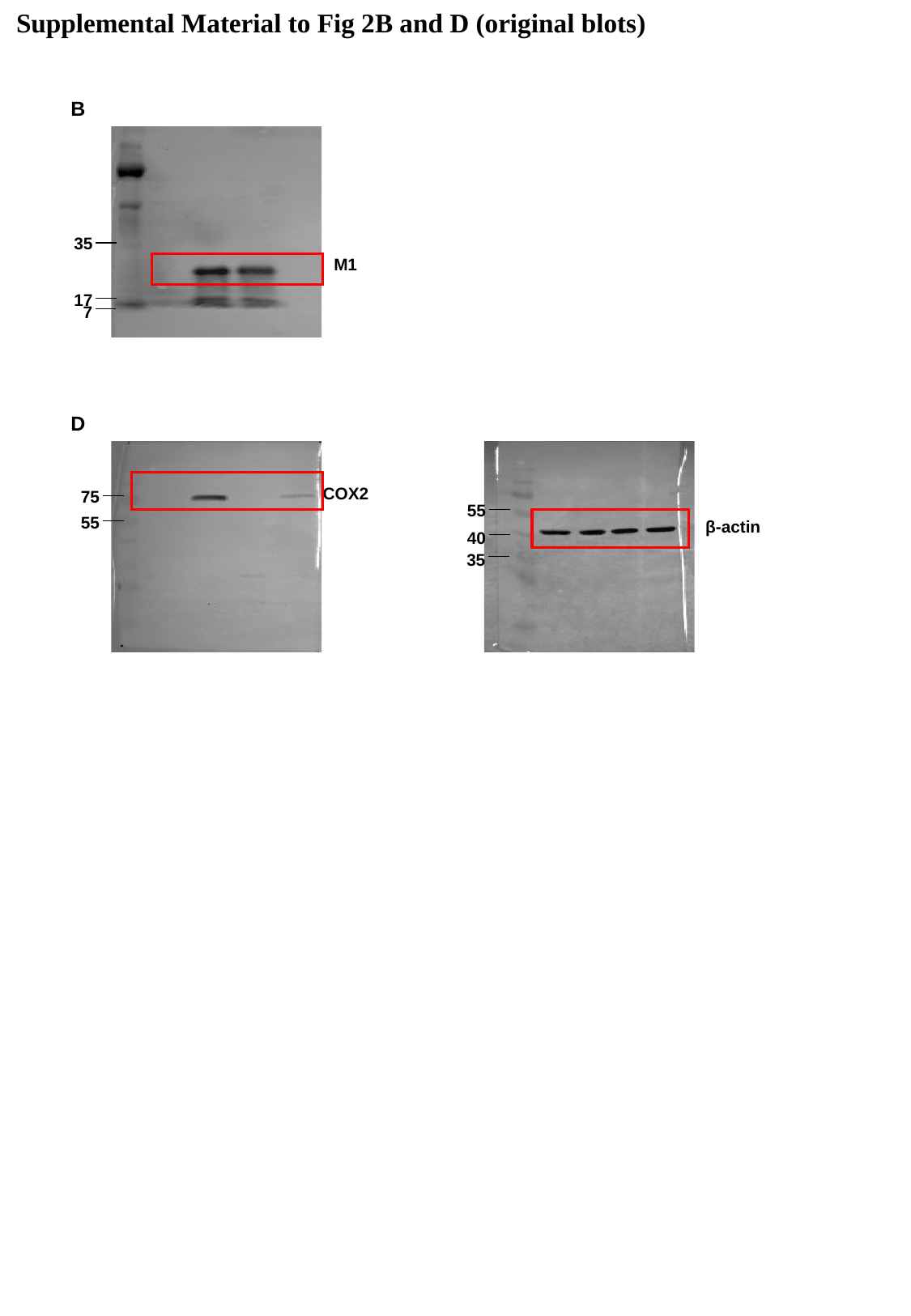

Supplemental Material to Fig 2B and D (original blots)
B
35
M1
17
7
D
COX2
75
55
55
β-actin
40
35

## Slide 6
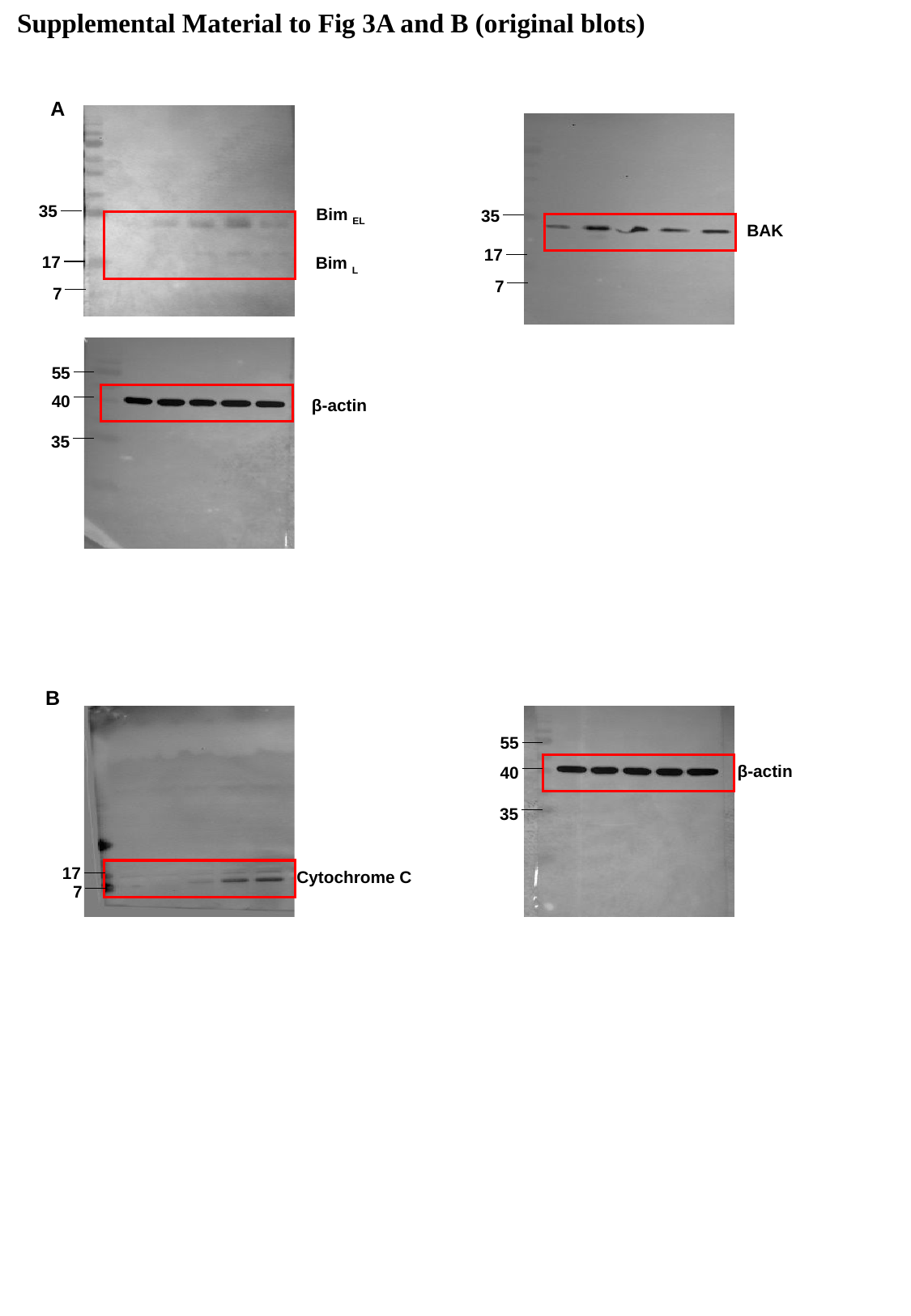

Supplemental Material to Fig 3A and B (original blots)
A
35
Bim EL
35
BAK
17
17
Bim L
7
7
55
40
β-actin
35
B
55
β-actin
40
35
17
Cytochrome C
7

## Slide 7
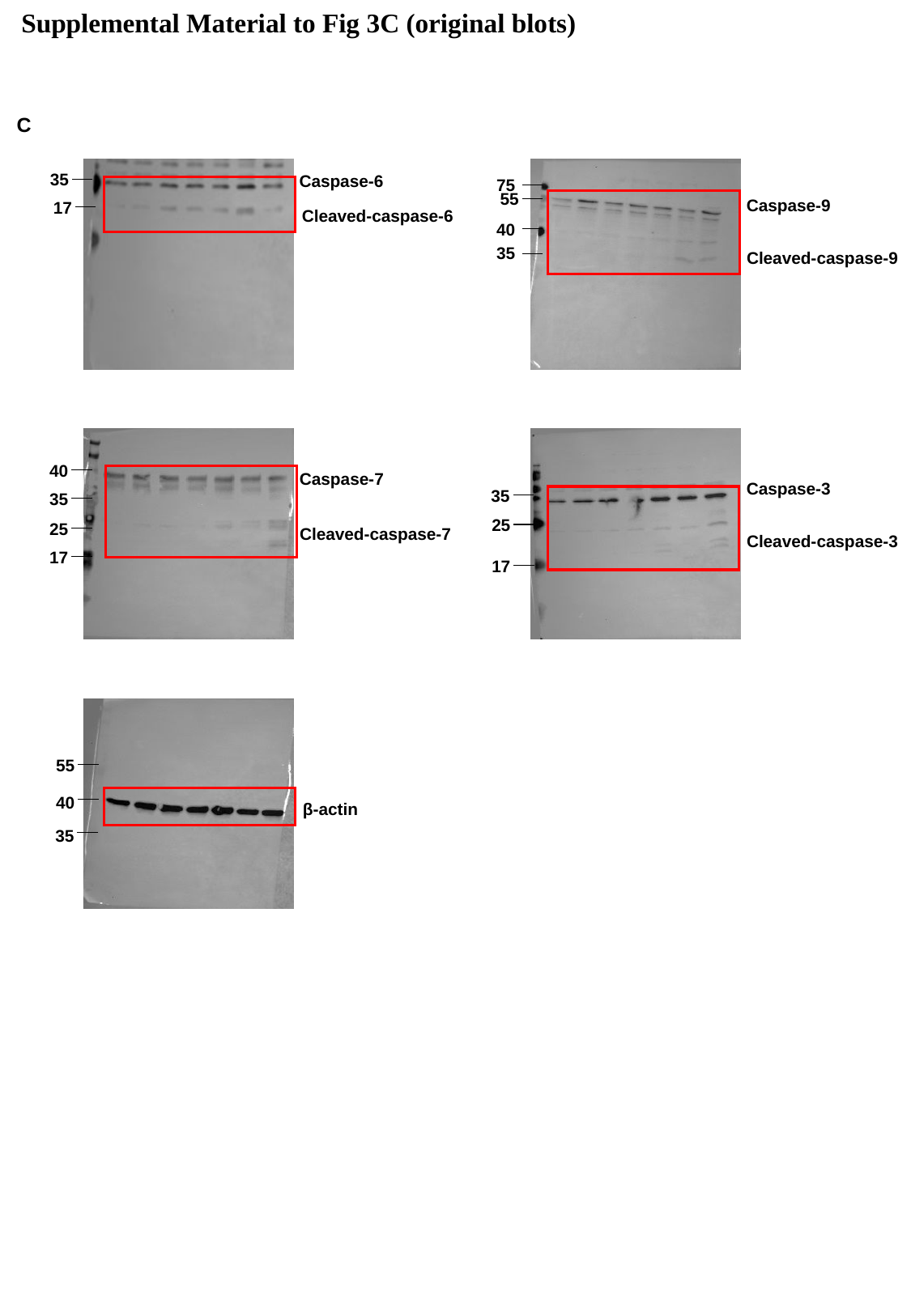

Supplemental Material to Fig 3C (original blots)
C
Caspase-6
Cleaved-caspase-6
Caspase-9
Cleaved-caspase-9
35
75
55
17
40
35
Caspase-7
Cleaved-caspase-7
Caspase-3
Cleaved-caspase-3
40
35
35
25
25
17
17
β-actin
55
40
35

## Slide 8
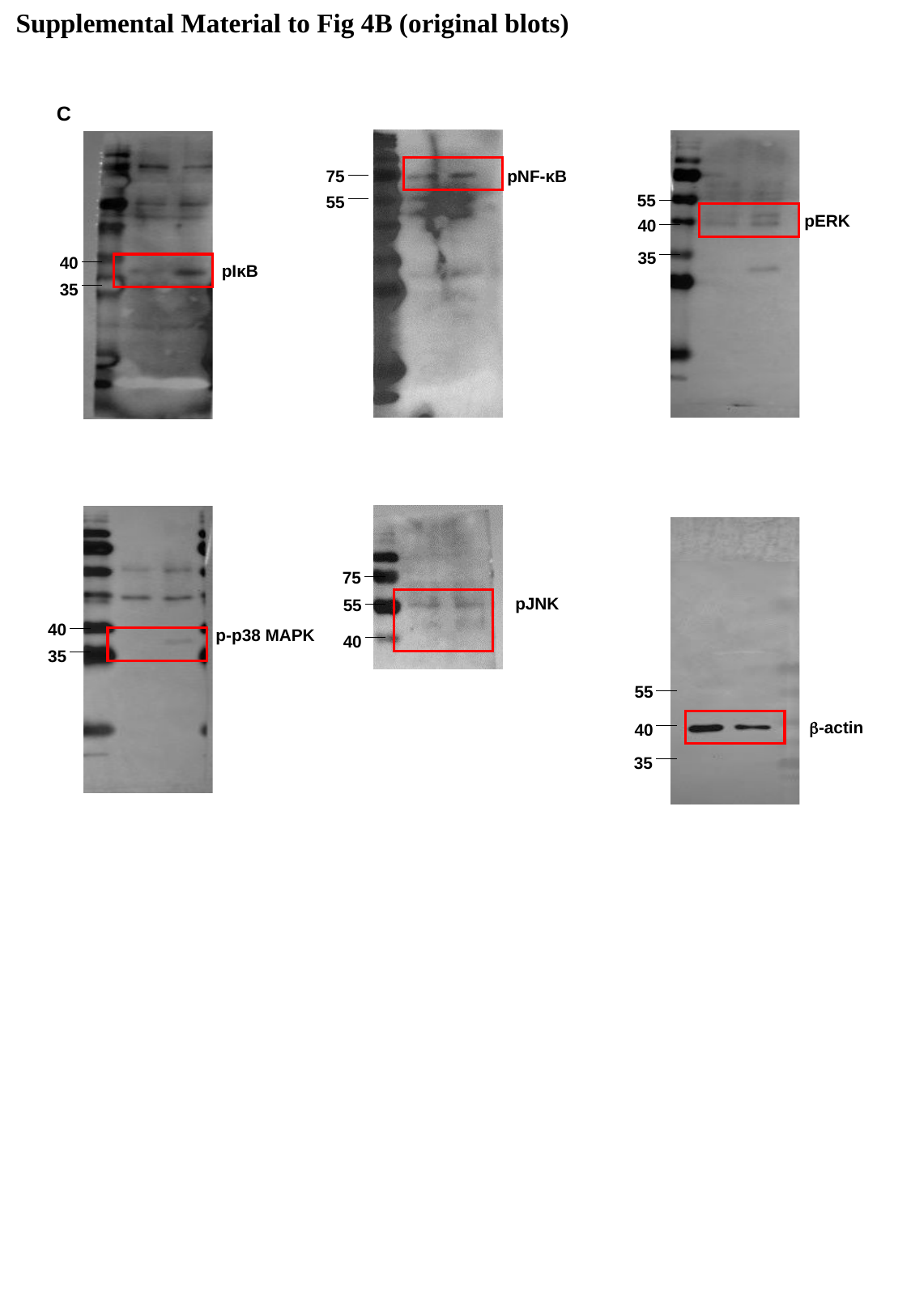

Supplemental Material to Fig 4B (original blots)
C
75
pNF-κB
55
55
pERK
40
35
40
pIκB
35
75
pJNK
55
40
p-p38 MAPK
40
35
55
b-actin
40
35

## Slide 9
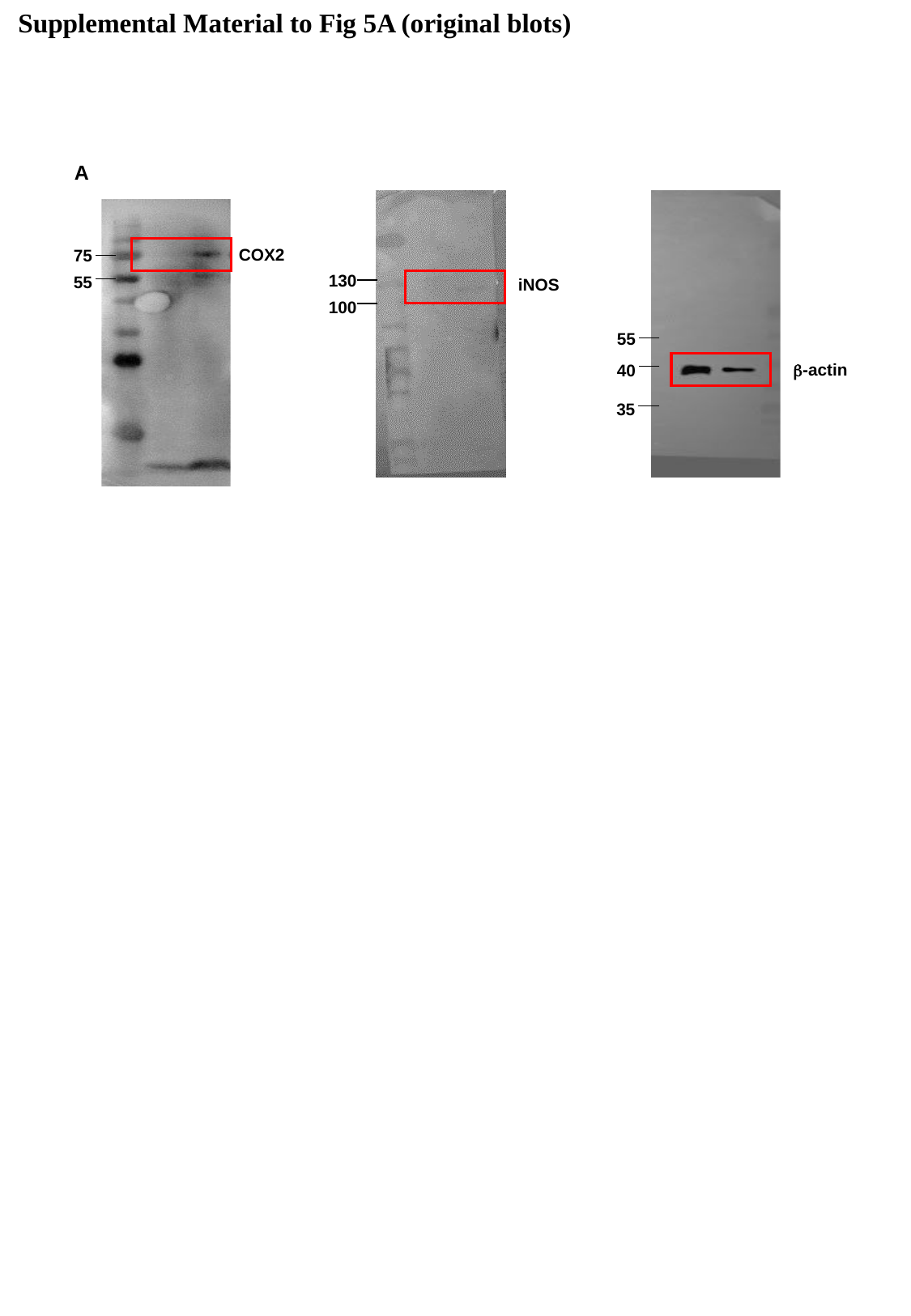

Supplemental Material to Fig 5A (original blots)
A
iNOS
b-actin
COX2
75
130
55
100
55
40
35

## Slide 10
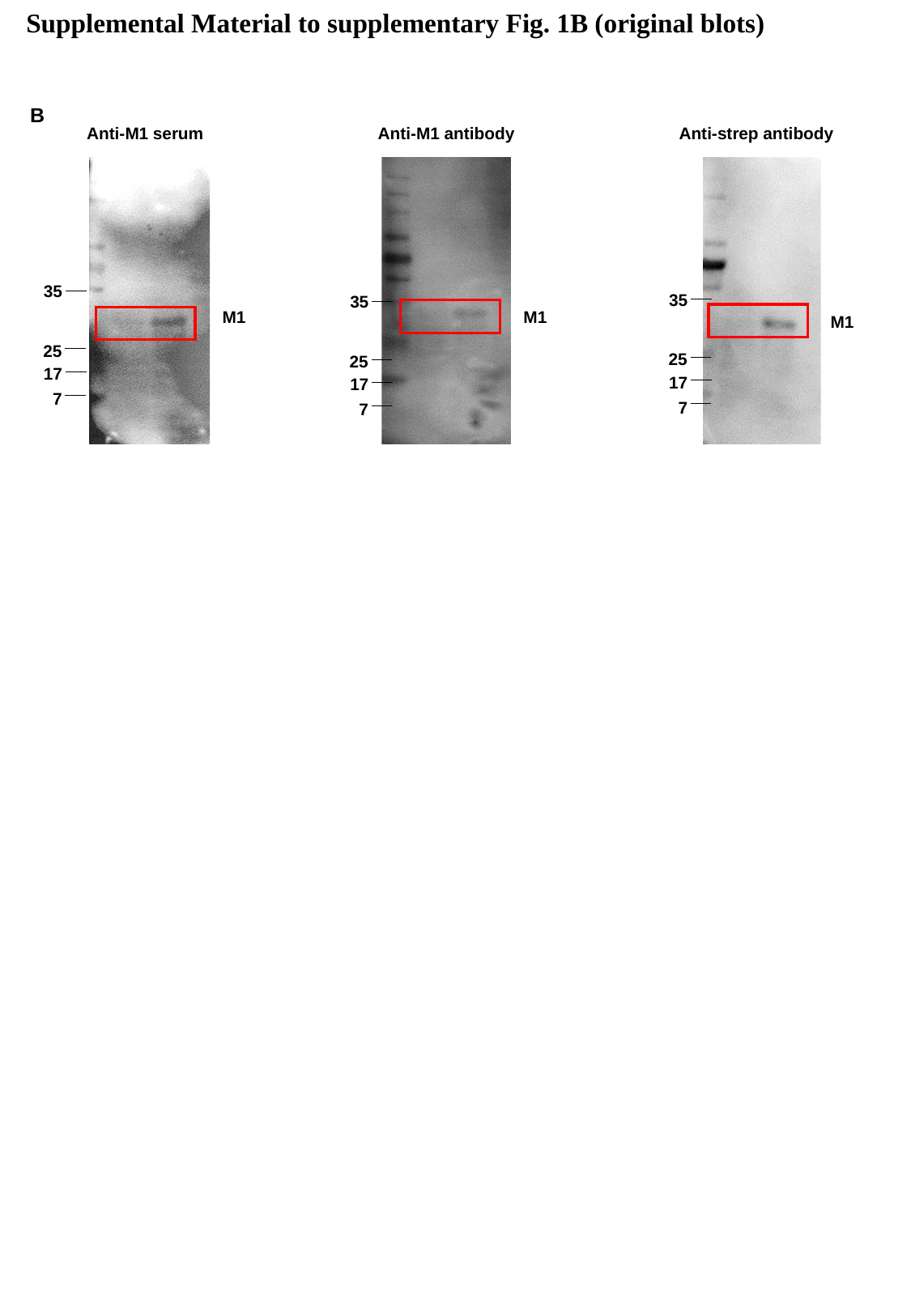

Supplemental Material to supplementary Fig. 1B (original blots)
B
Anti-M1 serum
Anti-M1 antibody
Anti-strep antibody
35
35
35
M1
M1
M1
25
25
25
17
17
17
7
7
7
